# C-Type Natriuretic Peptide Preserves Vascular and Cardiac Function in Sepsis

**DOI:** 10.1161/HYPERTENSIONAHA.125.25938

**Published:** 2026-02-04

**Authors:** Amie J. Moyes, Claire Sand, Leanne Young, Cristina Pérez-Ternero, Aemun T. Salam, Reshma S. Baliga, Shireen Mohammad, David B. Antcliffe, Anthony C. Gordon, Aisah A. Aubdool, Adrian J. Hobbs

**Affiliations:** 1 Faculty of Medicine and Dentistry, William Harvey Research Institute, Barts & The London Hospitals, Queen Mary University of London, United Kingdom (A.J.M., C.S., L.Y., C.P.-T., A.T.S., R.S.B., S.M., A.A.A., A.J.H.).; 2 Faculty of Medicine, Division of Anesthetics, Pain Medicine and Intensive Care, Imperial College London, United Kingdom (D.B.A., A.C.G.).

**Keywords:** arterial pressure, hypotension, lipopolysaccharides, natriuretic peptide, C-type, sepsis

## Abstract

**BACKGROUND:**

Sepsis is a life-threatening condition and a major cause of mortality in intensive care units worldwide, a clear unmet medical need. CNP (C-type natriuretic peptide) regulates inflammation and cardiovascular homeostasis, but its involvement in sepsis pathogenesis is not fully elucidated. This study investigated the intrinsic role of CNP, and therapeutic potential of the peptide, in offsetting the pathogenesis of sepsis.

**METHODS:**

Plasma concentrations of CNP, and its N-terminal cleavage product NT-proCNP (N-terminal pro-CNP), were measured in sepsis patients. Cardiac function, vascular hemodynamics, endothelial integrity, and biomarkers of inflammation were analyzed in wild-type, endothelium-restricted (ecCNP^−/−^), or cardiomyocyte-restricted (cmCNP^−/−^) CNP knockout animals, or global NPR (natriuretic peptide receptor)-C^−/−^ deficient mice, in etiologically distinct models of sepsis. CNP (0.2 mg/kg per d) was infused to rescue any adverse phenotype and probe therapeutic potential.

**RESULTS:**

Circulating [NT-proCNP] increased in sepsis patients and was associated with reduced disease severity. ecCNP^−/−^ mice exhibited an aggravated phenotype compared with wild-type mice in experimental sepsis, exemplified by impaired microcirculatory flow, edema, and increased expression of inflammatory biomarkers. In addition, cmCNP^−/−^ animals showed overt cardiac dysfunction following lipopolysaccharide treatment. This worsened phenotype was mirrored in NPR-C^−/−^ mice, implying that this cognate NPR subtype underpins the salutary actions of endogenous CNP. Pharmacological CNP administration improved microvascular perfusion, cardiac output, and inflammation in wild-type and ecCNP^−/−^, but not NPR-C^−/−^, mice.

**CONCLUSIONS:**

Endogenous CNP plays a protective role in sepsis by preserving microvascular perfusion, reducing inflammation, maintaining endothelial integrity, and sustaining cardiac function via NPR-C. Pharmacologically targeting CNP signaling warrants further evaluation as a potential therapeutic opportunity in sepsis.

**Novelty and Relevance**

**What Is New?**

Genetic deletion of CNP (C-type natriuretic peptide) exacerbates disease severity in experimental sepsis. Therapeutic administration of CNP markedly reduces sepsis pathogenesis via activation of cognate NPR (natriuretic peptide receptor)-C.

CNP release is increased in experimental sepsis and patients with the disorder; circulating [NT-proCNP] (N-terminal pro-C-type natriuretic peptide) correlates with lung hypoxemia.

**What Is Relevant?**

The multimodal, locally acting vasodilator, anti-inflammatory, and cardioprotective actions of CNP are an attractive target in sepsis where current therapeutics restore systemic pressure but fail to address microvascular dysfunction.

**Clinical/Pathophysiological Implications?**

Therapeutic interventions targeting CNP/NPR-C signaling warrant further investigation and evaluation as a tangible means to treat sepsis pharmacologically.

**See editorial by Sangaralingham and Burnett**

Sepsis is a leading cause of critical illness and mortality worldwide, yet the underlying pathobiology of this complex syndrome is poorly understood. The endothelium is a critical component of the host response to sepsis due to its role in regulating vascular tone, inflammation, thrombosis, and barrier function.^1^ Inflammatory mediators released during infection drive upregulation of vascular iNOS (inducible nitric oxide synthase), resulting in excessive NO production, which underlies the characteristic decrease in blood pressure in rodents and humans.^2,3^ This vascular insult also alters adhesion molecule expression, leukocyte migration, platelet aggregation, and vascular leak,^4,5^ which contributes to the diminished microcirculatory perfusion that can lead to organ failure.^6^ Notably, restoration of microcirculatory flow using vasodilator therapy improves tissue perfusion in animals and humans with sepsis.^7,8^ This highlights a contemporary paradox and therapeutic deficit in sepsis because guidelines advocate maintaining mean arterial blood pressure >65 mm Hg, but this is often achieved at the expense of preserving microcirculatory flow. The importance of endogenous vasodilators in maintaining tissue perfusion during sepsis is exemplified in trials using nitric oxide synthase (NOS) inhibitors which, despite having positive effects on blood pressure, caused more end-organ damage and mortality due to the disruption of microvascular homeostasis.^9,10^ This is supported by the positive pharmacological effect of nitrite, an NO precursor, in experimental sepsis.^11^ Therefore, there has been a push to identify novel therapeutic agents that can improve the microcirculatory dysfunction that underpins sepsis.

CNP (C-type natriuretic peptide) is a paracrine mediator that exerts similar cardioprotective effects to NO and plays a central homeostatic role in the cardiovascular system.^12–15^ One of the primary triggers of CNP release from the endothelium is exposure to proinflammatory mediators, which are established to play a role in the pathogenesis of sepsis.^16^ Indeed, sepsis is one of the few conditions in which elevated circulating CNP levels can be detected.^17,18^ However, relatively little is known about the (patho)physiological function(s) of CNP in sepsis, the cognate NPR (natriuretic peptide receptor) subtype (NPR-B or NPR-C) that mediates such roles, and the therapeutic potential of targeting this signaling pathway. Herein, we provide convincing mechanistic evidence addressing each of these uncertainties.

## Methods

### Animal Experimentation and Ethics

All animal procedures were conducted in accordance with the UK Home Office Animals (Scientific Procedures) Act of 1986, adhered to the Animal Research: Reporting of In Vivo Experiments guidelines, and were approved by a local animal welfare and ethical review board. Animals (both sexes, 16 weeks old, C57BL/6J background) were housed in a temperature-controlled environment with a 12-hour light-dark cycle. Food and water were accessible ad libitum. For in vivo experimentation, animals were randomly assigned to interventions, and the experimenter was blinded to treatment wherever feasible. For cell- and tissue-based studies, interventions were randomly assigned, but the experimenter was not blinded to treatment. Endothelial (ecCNP^−/−^) and cardiomyocyte (cmCNP^−/−^)-restricted CNP knockout mice were generated as previously described and phenotyped^12,14^; global NPR-C^19^ (NPR-C^−/−^) was the kind gift of Prof O. Smithies (University of North Carolina at Chapel Hill). Wild-type (WT or ^+/+^) littermates were used as controls.

Human plasma samples were procured from Imperial College Healthcare NHS Trust Tissue Bank, subcollection references ICHTB R17039 (septic samples) and SUR_DA_19_002 (nonseptic samples). The lung was the main source of infection for the patients with sepsis, and control (nonseptic) samples were obtained from intensive care unit (ICU) patients with no infection. Ethical approval was given by the Wales MREC (Ref: 12/WA/0196) and North London REC3 (Ref: 10/H0709/77). Patient demographics are provided in Table S1.

The authors declare that all supporting data are available within the article and the Supplemental Material.

### Biochemical Bioassays

Plasma concentrations of CNP and its N-terminal cleavage fragment, NT-proCNP (N-terminal pro-CNP), were determined by ELISA in samples from naive and lipopolysaccharide/cecal ligation and puncture (CLP) mice and patients with and without sepsis. NT-proCNP was measured using a commercially available colorimetric ELISA (Biomedica, Austria; BI-20812). Peptide extraction and centrifugal concentration (Speedvac, Thermo Scientific, United States) were required before performing the assay for CNP-22 in both mouse and human plasma. The extraction kit (Phoenix Pharmaceuticals, Germany; RK-EXTRACT) and protocol are described in the manual for the fluorescent CNP-22 ELISA kit (Phoenix Pharmaceuticals, Germany; FEK-012-03). Plasma nitrite (NO_2_^-^) plus nitrate (NO_3_^-^) concentrations (termed NO_x_) were measured as we have described previously using an established ozone-based chemiluminescence assay.^20^ Plasma ET-1 (endothelin-1) levels were measured by commercially available ELISA (R&D Systems, United Kingdom; DET-100).

### Models of Endotoxemia and Sepsis

Two well-validated, mechanistically distinct experimental models of endotoxemia/sepsis were used: administration of lipopolysaccharide (12.5 mg/kg, IP; 24-hour end point) or CLP (2 punctures with an 18G needle; 24-hour end point), as we and others have previously described.^2,20,21^ Mean arterial blood pressure and heart rate were monitored by radiotelemetry in conscious animals, and echocardiography was performed to assess heart structure and function, as we have previously described.^12,14^ Noninvasive laser Doppler imaging (Moor MK2 infrared-wavelength high-resolution scanner, Moor Instruments Ltd, Devon, United Kingdom) was used to assess blood flow in the ear and hindlimb in anaesthetized mice. Further details can be found in the Supplemental Material.

### Vascular Reactivity and Permeability

The aorta and mesenteric arteries from naive and lipopolysaccharide-treated animals were isolated, mounted, and normalized for organ bath pharmacology experiments, as previously described.^12^ A Miles assay was used to assess vascular extravasation as we have previously described.^22^ Further detail can be found in the Supplemental Material.

### Gene Expression

Standard biochemical and histochemical approaches were used to determine target gene and protein expression. Further details can be found in the Supplemental Material.

### Statistical Analysis

All data are expressed as mean±SEM, where n is the number of mice used in each experiment. Statistical analyses were performed using GraphPad Prism (version 10; GraphPad software, CA). For comparison of 2 groups of data, a 2-tailed, unpaired Student *t* test was used. A 2-way ANOVA was used to compare concentration-response curves and telemetry data. If ≥3 groups of data were compared, a 1- or 2-way ANOVA followed by an appropriate multiple comparisons test was used with adjustment for multiplicity. *P*<0.05 was considered statistically significant.

## Results

### Endothelium-Derived CNP Maintains Endothelial Function and Microcirculatory Perfusion During Endotoxemia

**Figure 1.**
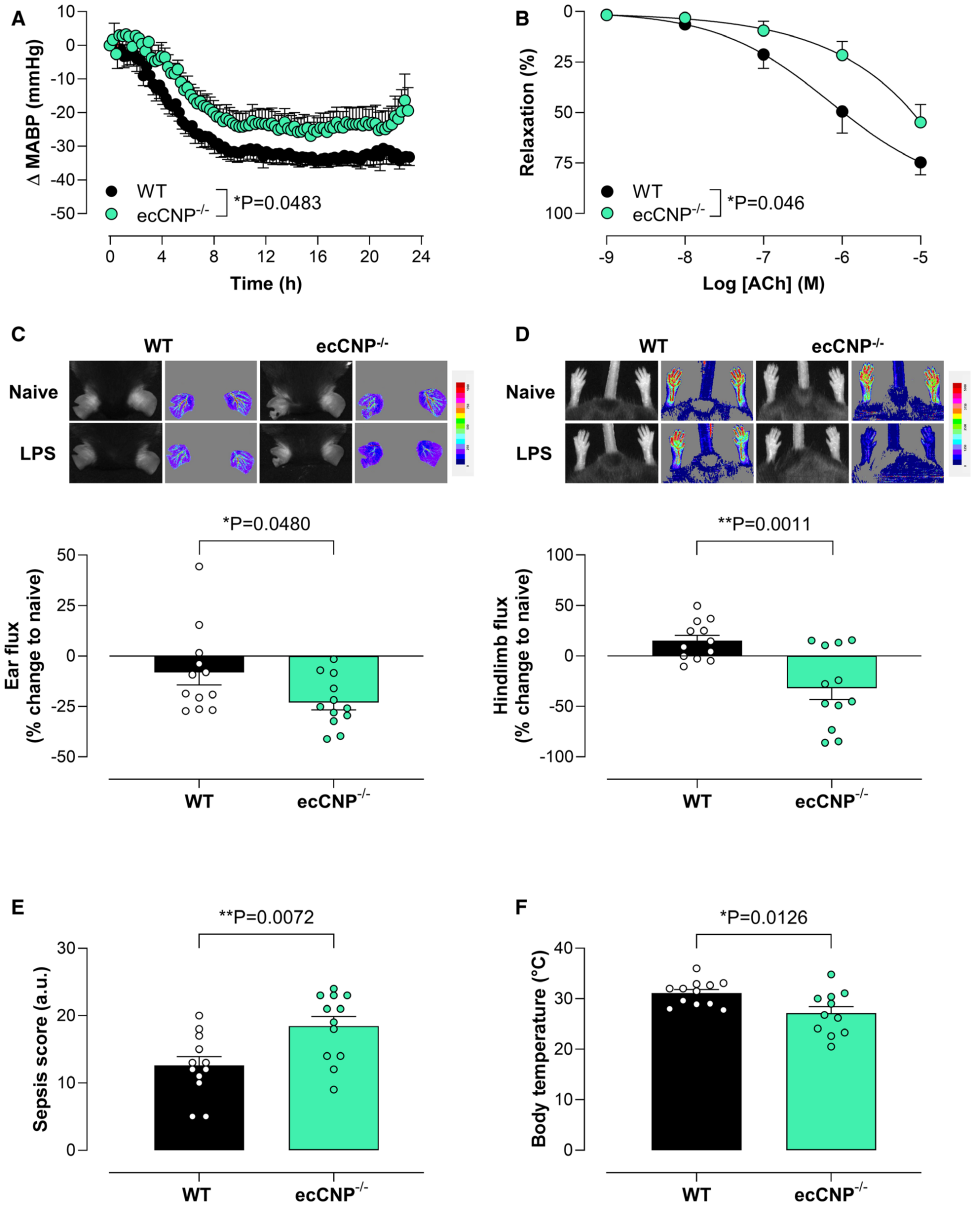
**Loss of endothelial-derived CNP (C-type natriuretic peptide) results in exacerbated severity of endotoxemia.** Change (Δ) in mean arterial blood pressure (MABP; **A**) and responses to the endothelium-dependent vasodilator, acetylcholine (ACh), in mesenteric arteries (**B**), and representative images and quantification (**C** and **D**) of blood flow in the ear and hindlimb (red=higher flow; blue=lower flow), sepsis score (**E**), and body temperature (**F**) 24 hours after lipopolysaccharide (LPS) administration (12.5 mg/kg, IP) in wild-type (WT) or endothelium-restricted CNP knockout (ecCNP^−/−^) mice. Data are represented as mean±SEM. n=12. Statistical analysis by 2-way ANOVA using a mixed effects model (**A** and **B**) or the unpaired Student *t* test (**C–F**). Each statistical comparison undertaken has an assigned *P* value (adjusted for multiplicity).

After lipopolysaccharide treatment, WT mice exhibited a greater decline in mean arterial blood pressure and a more rapid onset of hypotension compared with ecCNP^−/−^ animals (Figure 1A). Heart rate rose initially, consistent with reflex tachycardia, and then dropped overtly although the overall increase/decrease in heart rate in WT and ecCNP^−/−^ mice was similar (Figure S1A).

Mesenteric arteries isolated from ecCNP^−/−^ mice were significantly less responsive to the endothelium-dependent vasodilator acetylcholine in comparison to WT animals treated with lipopolysaccharide (Figure 1B). These differences in endothelium-dependent relaxation were not due to changes in contractile function (Figure S1B). Acetylcholine responses in mesenteric arteries from WT animals exposed to lipopolysaccharide were almost identical to vessels from naive animals (Figure S1C); however, the concentration-response curve to acetylcholine was shifted to the right in mesenteric arteries from ecCNP^−/−^ mice after treatment with lipopolysaccharide (Figure S1C). In contrast, acetylcholine responses were blunted in the aortas of animals treated with lipopolysaccharide in comparison to naive mice, but no significant differences were observed between the 2 genotypes (Figure S1E). Deletion of endothelial CNP had no effect on the contractile response of the aorta to 9,11-dideoxy-9α,11α-methanoepoxy PGF2α (prostaglandin F2alpha) or the vasorelaxant potency of the NO donor spermine-NONOate or ANP (atrial natriuretic peptide; Figure S1F through S1H).

### Endothelium-Derived CNP Maintains Microcirculatory Blood Flow During Endotoxemia

Endothelium-restricted CNP^−/−^ animals exhibited a significantly greater reduction in ear perfusion compared with WT mice following exposure to lipopolysaccharide (Figure 1C). This genotypic difference in the microcirculatory hypoperfusion, which typifies sepsis,^23^ was also observed in the hindlimb (Figure 1D). Baseline blood flow was comparable in both genotypes (Table S2). These hemodynamic changes were associated with increased disease severity as illustrated by a higher sepsis score and lower body temperature in ecCNP^−/−^ animals compared with WT littermates (Figure 1E and 1F).

### Endogenous CNP Dampens Inflammation During Endotoxemia

**Figure 2.**
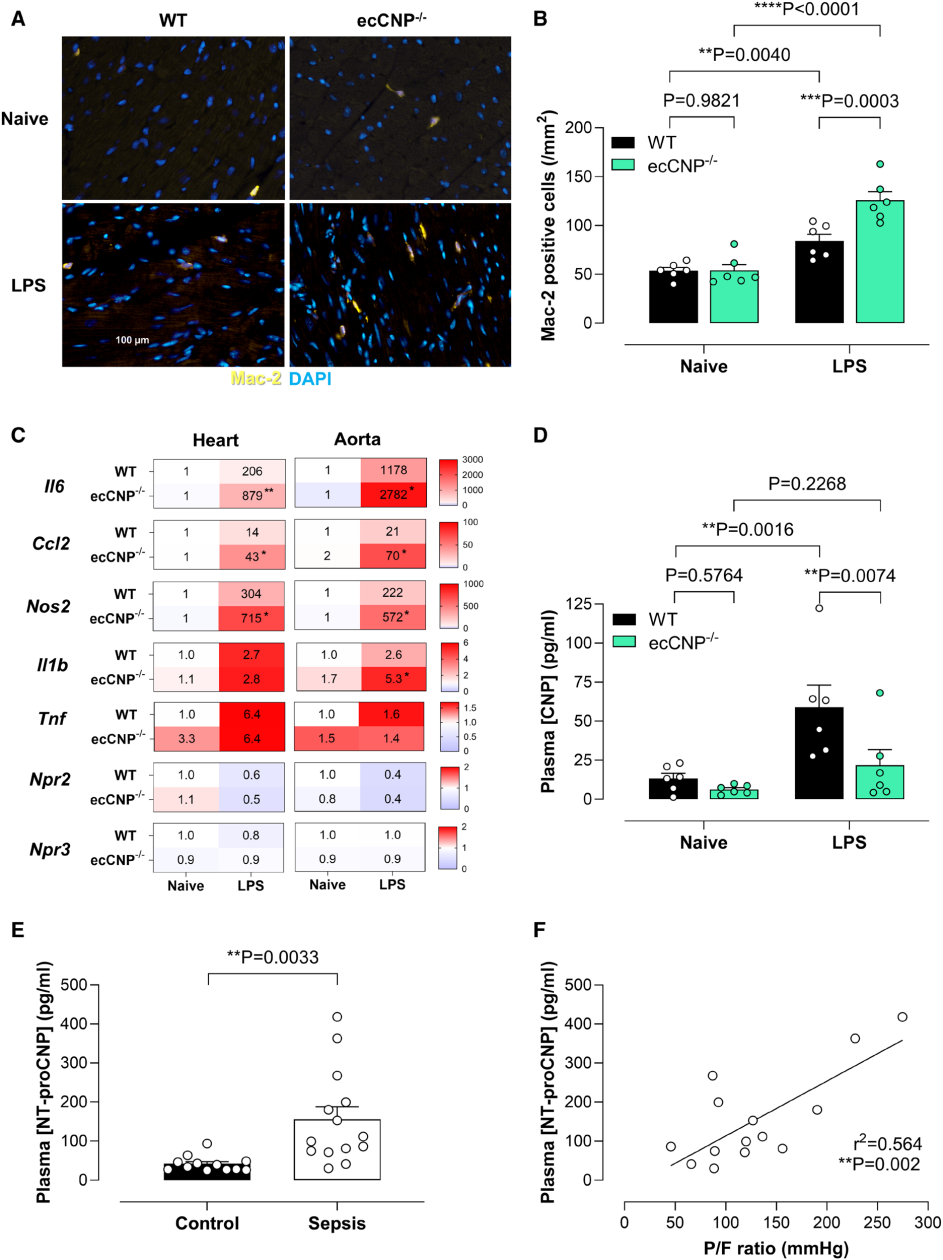
**Loss of CNP (C-type natriuretic peptide) results in greater macrophage infiltration and expression of inflammatory markers during endotoxemia, while CNP levels are raised in mice and patients with sepsis.** Representative immunostaining (**A**; macrophage marker Mac-2 [galectin-3], yellow; nuclei, 4′,6-diamidino-2-phenylindole [DAPI], blue) and quantification (**B**) of cardiac macrophage infiltration, mRNA expression of the inflammatory markers, C-C motif chemokine ligand 2 (CCL2 [monocyte chemotactic protein 1]), IL (interleukin)-6, iNOS (inducible nitric oxide synthase; Nos2), Il-1b, and tnf (tumor necrosis factor)-α, plus NPRs (natriuretic peptide receptors), NPR-B (*Npr2*) and NPR-C (*Npr3*), in the heart and aorta (**C**), and plasma CNP concentrations (**D**) in wild-type (WT) or endothelium-restricted CNP knockout (ecCNP^−/−^) mice in the absence and presence of lipopolysaccharide (LPS) administration (12.5 mg/kg, IP; 24 hours). Plasma concentrations of NT-proCNP (N-terminal pro-CNP; **E**) and positive correlation between plasma [NT-proCNP] and arterial oxygen pressure (PaO_2_)/fraction of inspired oxygen (FiO_2_) ratio (P/F ratio; **F**) in healthy controls and patients with sepsis. Data are represented as mean±SEM. n=6 to 14. Statistical analysis by 2-way ANOVA with the Fisher post hoc test (**B–D**), the unpaired Student *t* test (**E**), or simple linear regression analysis (**F**). Each statistical comparison undertaken has an assigned *P* value (adjusted for multiplicity).

The number of macrophages infiltrating the hearts of animals treated with lipopolysaccharide was significantly greater in ecCNP^−/−^ mice (Figure 2A and 2B). In accord, the expression of the inflammatory markers, IL (interleukin)-6, CCL2 (C-C motif chemokine ligand 2 or monocyte chemoattractant protein 1), Nos2 (iNOS), and IL-1β, was significantly elevated in lipopolysaccharide-treated ecCNP^−/−^ tissues in comparison to WT animals (Figure 2C; Figure S2A and S2B). NPR-B (*Npr2*) expression was reduced after lipopolysaccharide treatment, but there were no differences observed between ecCNP^−/−^ and WT mice (Figure 2C; Figure S2A and S2B). NPR-C (*Npr3*) mRNA expression was similar in both genotypes and was not altered by lipopolysaccharide (Figure 2C; Figure S2A and S2B). Hematoxylin and eosin staining also revealed small areas of focal myocytolysis in hearts from ecCNP^−/−^ animals treated, which was not observed in the hearts from WT mice (Figure S2C).

### Circulating CNP Concentrations Are Increased in Mice and Humans With Sepsis

In mice, lipopolysaccharide elicited a significant increase in circulating plasma [CNP] in WT animals, which was not observed in ecCNP^−/−^ mice (Figure 2D). CNP plasma levels in patients with sepsis were not different from nonseptic controls (Figure S3; it should be noted that the peptide levels were extremely low in both patient groups with a maximum of ≈3.5 pg/mL recorded in a patient with sepsis). NT-proCNP is the N-terminal fragment of the propeptide precursor of the biologically active form of CNP (CNP-22; measured above) and is considered a more robust marker of CNP biosynthesis because it is significantly less susceptible to enzymatic breakdown. NT-proCNP concentrations were almost 4-fold higher in plasma from patients with sepsis compared with nonseptic controls (Figure 2E), implying that there is increased CNP biosynthesis in sepsis, but levels of bioactive CNP-22 are not significantly raised. Moreover, there was a significant correlation between plasma [NT-proCNP] and the PO_2_ in arterial blood divided by the fraction of inspired oxygen (P/F ratio), a marker of lung hypoxemia and lung damage, with higher NT-proCNP levels associated with an improved P/F ratio (Figure 2F).

### NPR-C^−/−^ Animals Exhibit Reduced Endothelial Function and Diminished Microvascular Perfusion in Addition to Greater Inflammation After Endotoxin Challenge

**Figure 3.**
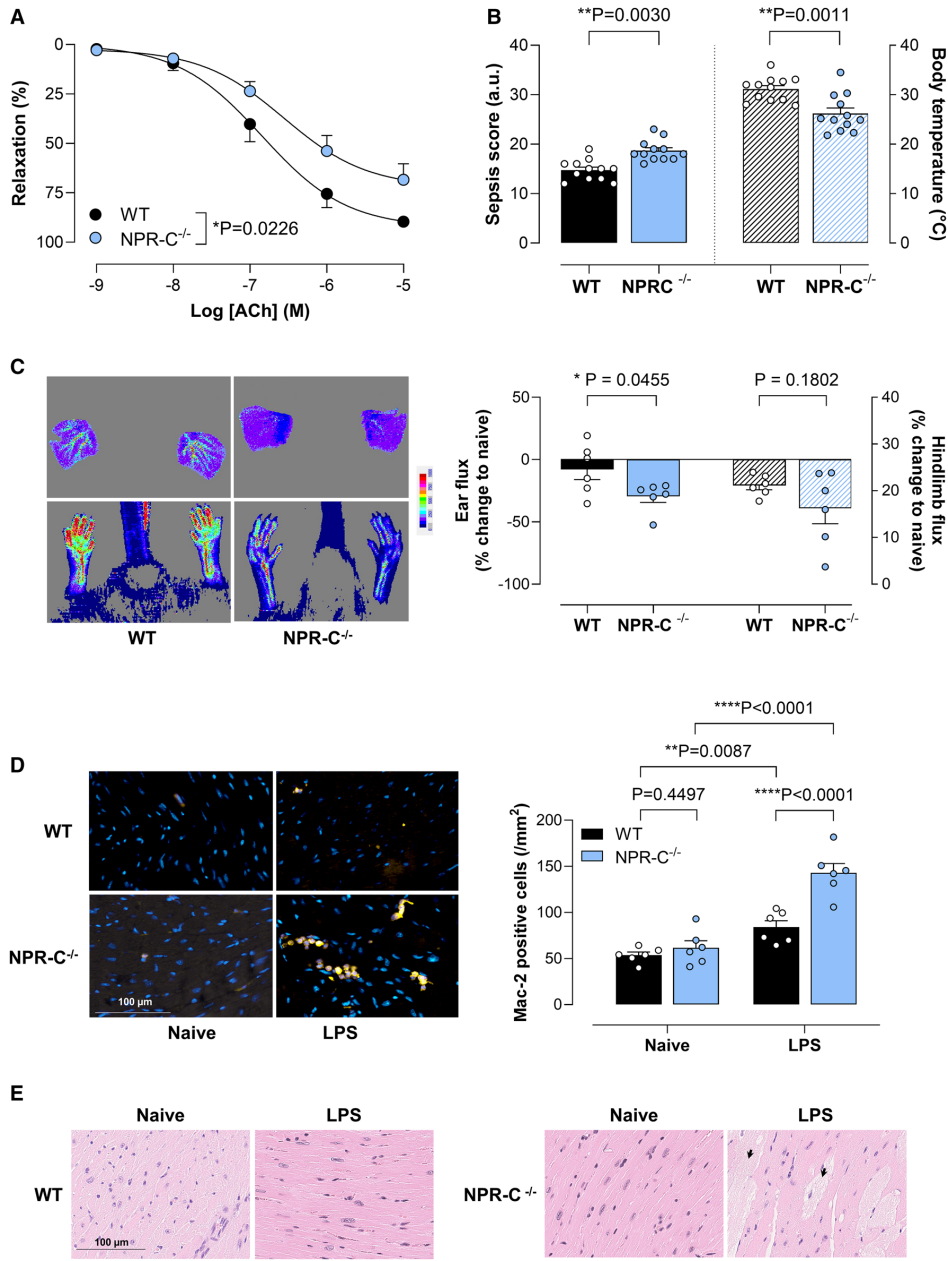
**Loss of NPR (natriuretic peptide receptor)-C results in exacerbated severity of endotoxemia.** Responses to the endothelium-dependent vasodilator, acetylcholine (ACh), in mesenteric arteries (**A**), sepsis score and body temperature (**B**), representative images and quantification of blood flow in the ear and hindlimb (red=higher flow; blue=lower flow; **C**), representative immunostaining (macrophage marker Mac-2 [galectin-3], yellow; nuclei, 4′,6-diamidino-2-phenylindole [DAPI], blue), quantification of aortic macrophage infiltration (**D**), and hematoxylin and eosin (H&E) staining of lipopolysaccharide (LPS)-treated hearts highlighting areas of myocytolysis (**E**; black arrows) 24 hours after LPS administration (12.5 mg/kg, IP) in wild-type (WT) or global NPR-C^−/−^ knockout mice. Data are represented as mean±SEM. n=6 to 12. Statistical analysis by 2-way ANOVA using a mixed effects model (**A**), unpaired Student *t* test (**B** and **C**), or 2-way ANOVA with the Fisher post hoc test (**D**). Each statistical comparison undertaken has an assigned *P* value (adjusted for multiplicity).

The resistance vessels of NPR-C^−/−^ animals, compared with arteries from WT littermates, displayed a blunted response to acetylcholine following lipopolysaccharide exposure (Figure 3A), mirroring the endothelial dysfunction observed in ecCNP^−/−^ mice (although NPR-C^−/−^ mice exhibited similar hemodynamic and heart rate changes to WT animals; Figure S4A and S4B). Mesenteric arteries from NPR-C^−/−^ animals did not show a change in vasoconstrictor sensitivity (Figure S4C). NPR-C^−/−^ mice also showed an essentially identical adverse phenotype to ecCNP^−/−^ animals when treated with lipopolysaccharide in terms of sepsis score (Figure 3B), body temperature (Figure 3B), and microcirculatory perfusion (ear and paw blood flow; Figure 3C). The NPR-C^−/−^ animals also exhibited more macrophage infiltration in the heart (Figure 3D), and the extent of myocytolysis was greater with larger, more numerous patches of damaged myocardium (Figure 3E); however, plasma levels of CNP in NPR-C^−/−^ animals treated with lipopolysaccharide were not different to naive mice (Figure S4D). The increased immune cell infiltrate into the heart was likely driven by higher expression of IL-6 and CCL2 (Figure S5A and S5B). The exacerbated microvascular dysfunction and inflammation in ecCNP^−/−^ and NPR-C^−/−^ mice during endotoxemia additionally manifested as exaggerated renal injury. Thus, endothelium-restricted deletion of CNP and global deletion of NPR-C resulted in a higher glomerular injury score in comparison to WT littermates (Figure S6A and S6B).

Neither endothelium-restricted deletion of CNP nor global deletion of NPR-C had any significant effect on ET-1 concentrations in the absence or presence of lipopolysaccharide. Plasma [NO_x_] was unaltered in ecCNP^−/−^ animals and only marginally elevated in NPR-C^−/−^ (Figure S7A through S7D).

### CNP Derived From Cardiomyocytes Contributes to the Regulation of Diastolic Function in Sepsis

Cardiac function deteriorated to a similar degree in WT and ecCNP^−/−^ mice treated with lipopolysaccharide (Figure S8A through S8D). Our previous work suggests that CNP released by cardiomyocytes plays an important role during cardiac stress in the setting of heart failure^14^; thus, we also investigated the contribution of cardiomyocyte-derived CNP (cmCNP) in endotoxemia. Indeed, while the ejection fraction and cardiac output of cmCNP^−/−^ animals did not differ in comparison to WT littermates (Figure S8E and S8F), diastolic function was significantly impaired (Figure S9A through S9C). Ear and hindlimb perfusion, sepsis score, and endothelial function of cmCNP^−/−^ mice were similar to those of WT animals (Figure S8G through S8I). An essentially identical phenotype was observed in NPR-C^−/−^ mice following endotoxin challenge, with significantly impaired diastolic function (Figure S9A, S9D, and S9E), whereas systolic function remained largely unaltered compared with WT (Figure S7J and S7K). These findings suggest that cardiomyocyte-derived CNP, acting via NPR-C, protects the heart from sepsis-induced diastolic dysfunction.

### Animals Lacking Endothelial CNP or NPR-C Exhibit a Worse Phenotype When Subjected to CLP

**Figure 4.**
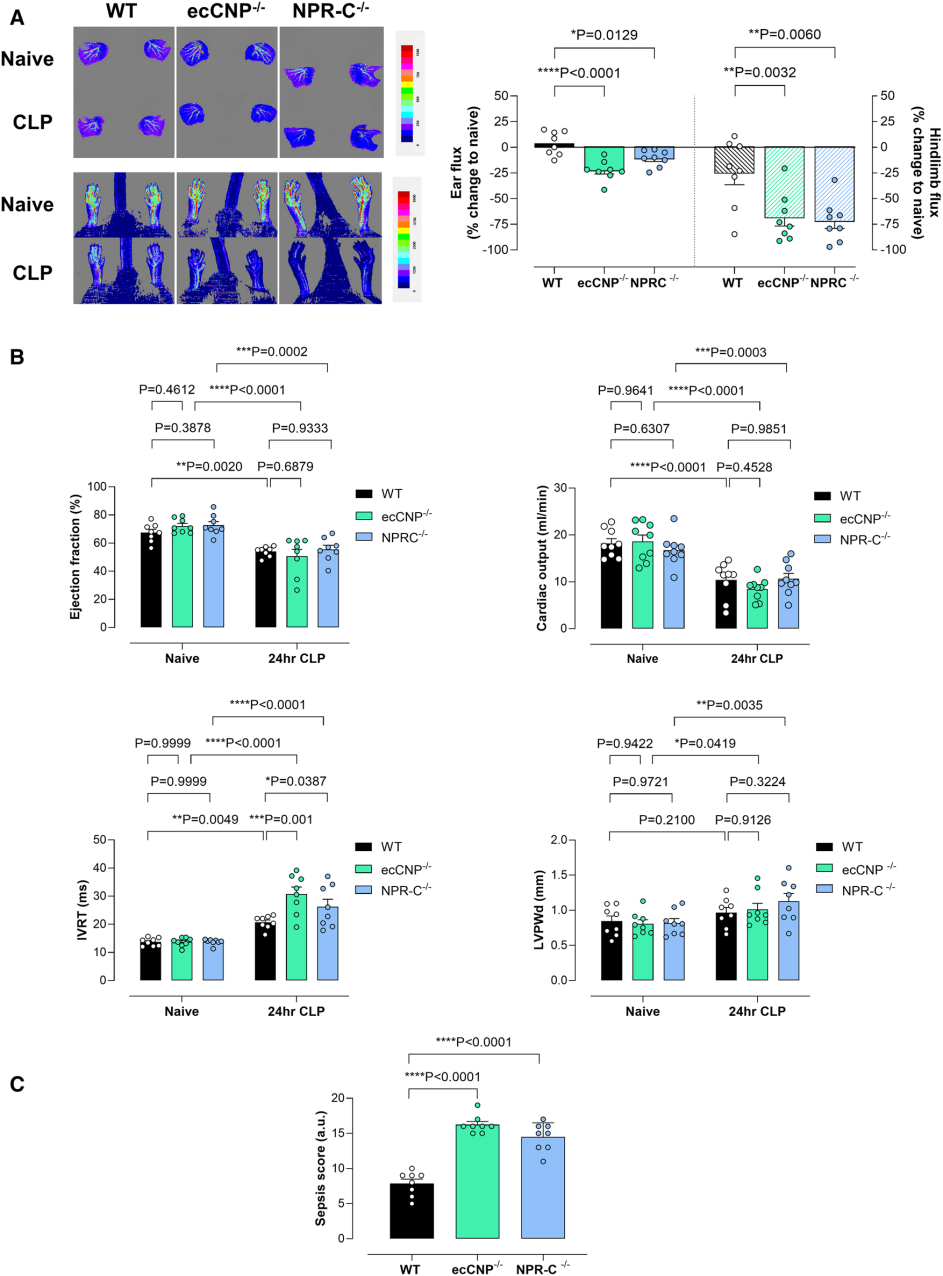
**Loss of endothelium-derived CNP (C-type natriuretic peptide) or NPR (natriuretic peptide receptor)-C results in increased sepsis severity following cecal ligation and puncture (CLP).** Change in ear and hindlimb perfusion (red=higher flow; blue=lower flow; **A**), indices of cardiac structure and function (**B**; ejection fraction, cardiac output, isovolumic relaxation time [IVRT], and left ventricular posterior wall diameter at diastole [LVPWDd]), and sepsis score (**C**) in wild-type (WT), endothelium-restricted CNP (ecCNP^−/−^), or NPR-C^−/−^ knockout mice in the absence (naive) and presence of CLP (2 punctures with an 18G needle; 24 hours). Data are represented as mean±SEM. n=8. Statistical analysis by 1-way ANOVA with the Dunnett post hoc test (**A**), 2-way ANOVA with the Tukey post hoc test (**B**), or 1-way ANOVA with the Šidák post hoc test (**C**). Each statistical comparison undertaken has an assigned *P* value (adjusted for multiplicity).

We performed CLP to demonstrate that the findings from the endotoxin model could be recapitulated in a model that more closely resembles human infection and also incorporates an element of resuscitation. Akin to animals treated with lipopolysaccharide, both ecCNP^−/−^ and NPR-C^−/−^ subjected to CLP exhibited reduced perfusion of the ear and hindlimb compared with WT littermates (Figure 4A). Systolic cardiac function following CLP was similar between genotypes; however, diastolic function was impaired in ecCNP^−/−^ and NPR-C^−/−^ in comparison to WT mice (Figure 4B). An increase in ventricular wall diameter was also observed in ecCNP^−/−^ and NPR-C^−/−^ animals, which did not occur in WT mice (Figure 4B). A higher sepsis score was also apparent in both knockout strains (Figure 4C).

### Loss of Endothelial CNP or NPR-C Increases Cardiac Edema and Vascular Permeability During Endotoxemia

**Figure 5.**
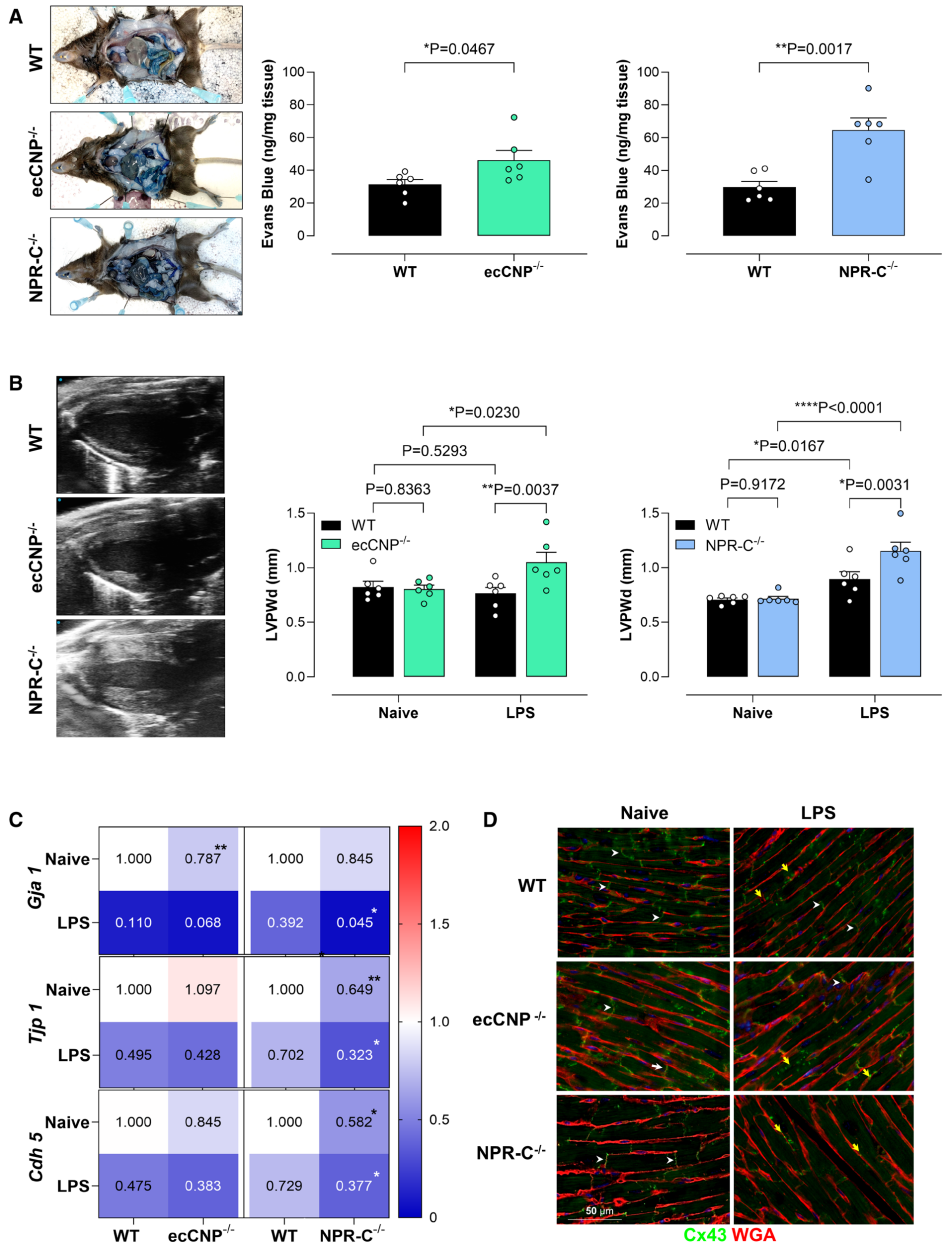
**Loss of endothelium-derived CNP (C-type natriuretic peptide) or NPR (natriuretic peptide receptor)-C results in endothelial barrier function, disarrangement of junctional proteins, and cardiac edema during endotoxemia.** Representative images and quantification of Evans blue extravasation (**A**), representative b-mode images and quantification of left ventricular posterior wall diameter (LVPWd; **B**), mRNA expression of the junctional proteins Cx43 (connexin 43; *Gja 1*), ZO (zonula occluden)–1 (*Tjp1*), and vascular endothelial cadherin (VE-cadherin; *Cdh 5*; **C**), in wild-type (WT), endothelium-restricted CNP knockout (ecCNP^−/−^), or global NPR-C^−/−^ knockout mice in the absence (naive) or presence of lipopolysaccharide (LPS) administration (12.5 mg/kg, IP; 24 hours). Representative images of Cx43 immunostaining (Cx43, green; cell membrane marker wheat germ agglutinin [WGA], red) in the hearts of naive and LPS-treated NPR-C^−/−^ mice (**D**). White arrows indicate Cx43 localization at the intercalated disk of cardiomyocytes; yellow arrows indicate expression at the lateral edges of the cells. Data are represented as mean±SEM. n=6. Statistical analysis by the unpaired Student *t* test (**A**) or 2-way ANOVA with the Fisher post hoc test (**B** and **C**). Each statistical comparison undertaken has an assigned *P* value (adjusted for multiplicity).

Cardiac edema is known to be an underlying cause of diastolic dysfunction^24,25^; therefore, we investigated whether vascular permeability was altered in the knockout strains. Greater extravasation was observed in the hearts of ecCNP^−/−^ and NPR-C^−/−^ in comparison to WT animals (Figure 5A). Interestingly, there was a dramatic increase in left ventricular wall diameter consistent with cardiac edema (particularly because myocardial remodeling is unlikely in the 24-hour time course of this model) in ecCNP^−/−^ and NPR-C^−/−^ mice (Figure 5B). This pathogenic cardiac phenotype is observed in septic animals and patients.^26^

We also examined the expression of junctional proteins known to regulate barrier integrity. *Gja 1* (Cx43 [connexin 43]), *Tjp 1* (ZO-1 [zonula occluden-1]), and *Cdh 5* (VE [vascular endothelial]-cadherin) gene expressions were reduced in the heart after exposure to lipopolysaccharide (Figure 5C; Figure S10). In fact, the expression of Cx43 was lower in naive ecCNP^−/−^ animals. Expression of Cx43 and ZO-1 was also reduced in NPR-C^−/−^ treated with lipopolysaccharide. In addition, NPR-C^−/−^ mice exhibited lower expression of VE-cadherin at baseline and following lipopolysaccharide treatment (Figure 5C; Figure S10).

Distinct localization of Cx43 was observed at the intercalated disks of cardiomyocytes and blood vessels in the heart (Figure 5D). Treatment with lipopolysaccharide induced redistribution of Cx43 to the lateral borders of the cardiomyocytes, a phenomenon that is observed in failing hearts.^27^ After lipopolysaccharide, some expression of Cx43 was still observed at the intercalated disk in WT animals and ecCNP^−/−^ mice, but little immunostaining was observed in NPR-C^−/−^ mice, most of which was lateralized.

### Treatment With CNP Improves Peripheral Blood Flow and Cardiac Function, and Reduces Inflammation in Endotoxemia

**Figure 6.**
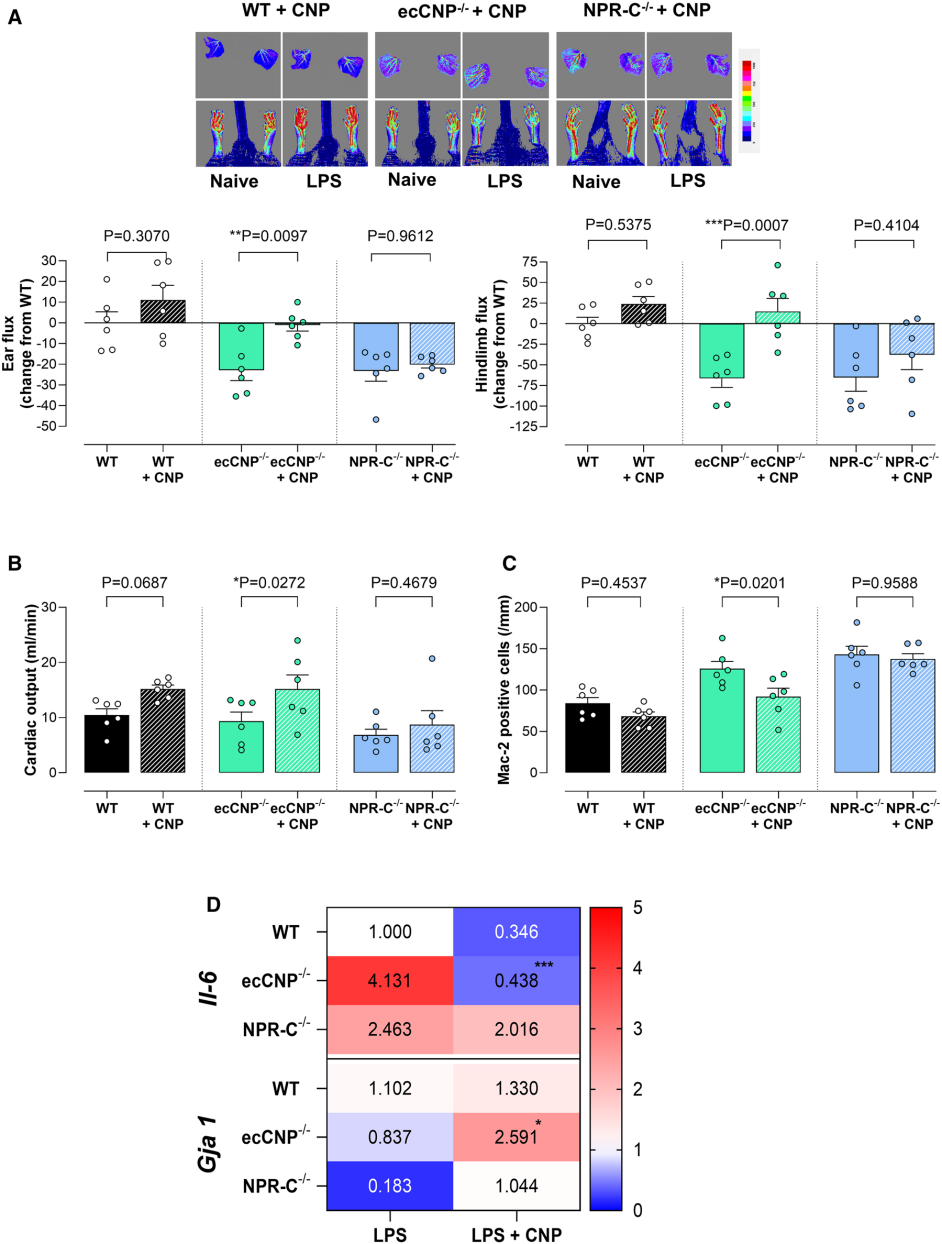
**CNP (C-type natriuretic peptide) infusion improves microcirculatory blood flow, cardiac function and reduces inflammation in endotoxemia.** Representative images and quantification of blood flow in the ear and hindlimb (red=higher flow; blue=lower flow; **A**), cardiac output (**B**), macrophage infiltration (Mac-2 [galectin-3] positive cells; **C**), and mRNA expression of IL (interleukin)-6 and connexin 43 (*Gja 1*; **D**), 24 hours after lipopolysaccharide (LPS) administration (12.5 mg/kg, IP) in wild-type (WT), endothelium-restricted CNP knockout (ecCNP^−/−^), or global NPR-C^−/−^ knockout mice in the absence and presence of CNP infusion (0.2 mg/kg per d, SC). Data are represented as mean±SEM. n=6. Statistical analysis by 1-way ANOVA with the Šidák post hoc test. Each statistical comparison undertaken has an assigned *P* value (adjusted for multiplicity).

CNP infusion improved ear and hindlimb blood flow in ecCNP^−/−^ animals (Figure 6A). Indices of cardiac function were also enhanced in ecCNP^−/−^ animals upon treatment with CNP; cardiac output increased (Figure 6B), and there was a decrease in isovolumic relaxation time (Figure S11A). CNP reduced macrophage infiltration in ecCNP^−/−^ mice (Figure 6C); this was accompanied by a decrease in the expression of the inflammatory cytokine IL-6 (Figure 6D; Figure S11B). Cx43 (*Gja 1*) expression was significantly increased in ecCNP^−/−^ treated with CNP (Figure 6D; Figure S11C), and less lateralization could be observed at the intercalated disk of WT and ecCNP^−/−^, akin to naive animals (Figure S11D). In contrast, and critical to the understanding of the cognate receptor underpinning the beneficial actions of CNP, peripheral perfusion, cardiac function, macrophage infiltration, inflammation, and connexin expression/localization were not altered by CNP administration in NPR-C^−/−^ animals (Figure 6A through 6D; Figure S11A through S11D), confirming CNP/NPR-C signaling as the central salutary pathway.

## Discussion

One of the major challenges in the management of sepsis is maintaining hemodynamic coherence; that is, improving both systemic pressure and microcirculatory flow to preserve organ perfusion.^28^ Thus, understanding the endogenous mechanisms that regulate and coordinate cardiovascular homeostasis and maintain this delicate balance during sepsis may aid the development of new therapeutics. Herein, we establish that a loss of endothelial CNP results in a more severe phenotype in sepsis compared with WT animals despite greater preservation of mean arterial blood pressure; this manifests as endothelial dysfunction, microcirculatory hypoperfusion, immune cell infiltration, renal damage, and edema. There is also a contribution from CNP of cardiomyocyte origin in the cardiac dysfunction characteristic of endotoxemia. The exacerbated pathogenesis is recapitulated in mice lacking NPR-C, suggesting that it is this cognate receptor that largely underlies the beneficial actions of CNP. In addition, we demonstrate that pharmacological administration of CNP offsets the pathogenesis of sepsis.

The importance of CNP as an endothelium-derived vasodilator increases inversely to blood vessel diameter, supporting the role of the peptide as an EDHF (endothelium-derived hyperpolarizing factor) in the microcirculation.^12^ Indeed, the vascular dysfunction in experimental sepsis identified herein is greater in resistance compared with conduit arteries and in ecCNP^−/−^ versus WT mice, implying that changes in EDHF bioactivity might underpin this phenomenon. Interestingly, NPR-C^−/−^ mice exhibit a similar phenotype to ecCNP^−/−^ animals with impaired vasodilation and peripheral perfusion during sepsis. However, the blood pressure of these animals was similar to that of WT mice treated with lipopolysaccharide, unlike ecCNP^−/−^ animals. This may be due to the dual capacity of this receptor to (1) signal and (2) clear natriuretic peptides,^19^ thereby accentuating the hypotensive actions of ANP and BNP (brain natriuretic peptide). In addition, we did not observe any overt sex differences in the vascular or cardiac complications exhibited by ecCNP^−/−^ or NPR-C^−/−^ animals exposed to lipopolysaccharide or CLP although the study was not adequately powered to permit prospective analyses of any sex difference(s). Therefore, further work in this area would need to be undertaken to determine whether this might be apparent.

CNP also appears to preserve the diastolic function of the heart of mice exposed to lipopolysaccharide, which fits with previous work showing positive lusitropic effects of CNP in both normal and failing hearts.^29,30^ The striking increase in LV wall diameter observed in the knockout animals is similar to the edema (albeit difficult to discern whether myocardial or pericardial) reported in sepsis, myocarditis, COVID-19, and ischemia-reperfusion injury.^26,31^ An increase in vascular leak due to disruption of tight and gap junctions has also been shown to induce diastolic dysfunction in mice, a pseudotamponade phenotype. This hints that the changes in cardiac morphology (ie, edema) and function observed in our study are linked.^25^ Global deletion of CNP is known to alter the distribution of the junctional protein ZO-1, resulting in dysfunction of the blood-brain barrier.^22^ ZO-1 also interacts with the gap junction Cx43^32^ and VE-cadherin,^33^ which are reduced in sepsis and associated with breakdown of the endothelial barrier.^34^ Remarkably, naive ecCNP^−/−^ and NPR-C^−/−^ animals exhibit reduced expression of ≥1 of these junctional proteins with a further reduction observed during endotoxemia. Indeed, previous work has shown that CNP reverses the downregulation of Cx43 induced by Ang II (angiotensin II) in rat atria via NPR-C.^35^ Cx43 can preserve the endothelial barrier via the formation of stabilizing networks with ZO-1, VE-cadherin, and the actin cytoskeleton.^36^ Therefore, it is possible that disruption of these complexes underlies the increase in permeability observed in mice lacking CNP or NPR-C. This process may be facilitated by the shedding effect of CNP on the glycocalyx.^37^ Cx43 is also highly expressed at the intercalated disk where it is involved in mechanical coupling and electrochemical communication.^27^ Our data indicate significant loss of this protein at the intercalated disk in septic NPR-C^−/−^ mice, suggesting that disruption of intercellular coupling might also contribute to worse myocardial function in these animals. We also explored the possibility that altered generation of NO or endothelin-1 (mediators known to contribute to sepsis pathogenesis^38,39^) in mice with cell-restricted deletion of CNP or global knockout of NPR-C might underpin some of the vascular and cardiac actions of CNP in sepsis. However, there were no overt changes in plasma NO_x_ or endothelin-1 across genotypes in the absence or presence of lipopolysaccharide.

Another cardioprotective function of CNP resides in its anti-inflammatory activity. Macrophage infiltration and markers of inflammation during endotoxemia were significantly elevated in ecCNP^−/−^ and NPR-C^−/−^ mice, suggesting that endogenous CNP signaling via NPR-C is important for dampening inflammation. Excessive inflammation in the heart can induce myocardial damage such as colliquative myocytolysis,^40^ which was observed histologically in both knockout animals. These observations fit with a recent report demonstrating the success of neprilysin and angiotensin receptor blockers in experimental autoimmune myocarditis, which was attributed to augmented circulating CNP levels and increased NPR-C activation.^41^

Herein, we also demonstrate the therapeutic utility of CNP (at a subhypotensive dose^14^) in improving both vascular and cardiac function. Indeed, the capacity of CNP to enhance microcirculatory perfusion and cardiac output independent of blood pressure is a key concept that supports the peptide as an intrinsic defense mechanism and potential therapeutic. Our observations also hint that NPR-C, rather than NPR-B, is the superior therapeutic target as expression of the former receptor is maintained during sepsis, whereas the latter is significantly reduced. Moreover, the beneficial pharmacological actions of CNP were not apparent in NPR-C^−/−^ animals, confirming the importance of this receptor subtype to the pathophysiology of sepsis. However, because we did not have access to animals with cell-restricted deletion of NPR-B, these data are only indirect evidence of the lack of involvement of this alternate cognate receptor subtype. Of note, pharmacological administration of CNP significantly improved indices of disease severity in ecCNP^−/−^ mice but offered a much more modest salutary effect in WT animals. This illustrates 2 key points. First, the intrinsic generation of CNP constitutes a host defense mechanism in sepsis. Second, therapeutic targeting of CNP signaling would be most efficacious in patients with sepsis whose endogenous CNP levels are low. The latter scenario appears to be true for the majority of cases because our findings indicate that circulating NT-proCNP levels are elevated in patients with sepsis, whereas the plasma concentrations of CNP are not. This supports the concept that CNP is released as a protective mechanism during sepsis, but levels of the biologically active peptide are suboptimal (possibly due to increased NEP [neutral endopeptidase (neprilysin)] activity). Accordingly, administration of a NEP inhibitor to prevent the inactivation of CNP may also hold therapeutic potential. Soluble NEP levels are elevated in patients with septic shock,^42^ which might explain why we observed that patients with sepsis had higher levels of plasma NT-proCNP (the byproduct of CNP bioactivation) in comparison to controls, whereas levels of biologically active CNP (CNP-22) remained low. This is in contrast to previous work exploring the role of ANP/BNP in sepsis, which reported that deletion of cognate NPR-A was shown to be beneficial rather than detrimental.^20^ In addition, we show that in patients with sepsis, circulating levels of NT-proCNP are inversely correlated with the PO_2_ in arterial blood (PaO_2_) divided by the fraction of inspired oxygen (FiO_2_), indicating that lower NT-proCNP concentrations are associated with a more severe hypoxemia and lung injury and greater disease severity (although we were not able to establish associations with harder outcomes such as mortality); these data corroborate earlier studies, indicating that the N-terminal fragment may offer potential as a diagnostic and prognostic biomarker.^43,44^ This observation implies that disrupted CNP signaling may be associated with worse clinical symptoms in this patient population and treatments which augment CNP might be of benefit.

## Perspectives

Our findings establish that endogenous CNP released in response to infection by endothelial cells and cardiomyocytes is important for regulating endothelial function, microcirculatory flow, cardiac function, barrier integrity, and inflammation. Therapeutic administration of CNP markedly improves microvascular perfusion and cardiac output, and dampens inflammation via activation of cognate NPR-C. As a whole, this work advocates further evaluation of interventions targeting CNP and NPR-C in the vasculature and heart during sepsis as a tangible means to treat the disease pharmacologically. The capacity of CNP/NPR-C-targeted therapy to increase microvascular perfusion and cardiac output without a significant drop in systemic blood pressure would integrate seamlessly with current sepsis treatments and, perhaps, reduce the need for vasopressors and inotropes. This approach would align with the recent findings from the ANDROMEDA-SHOCK-2 study^45^ in which personalized hemodynamic resuscitation targeting microvascular perfusion status afforded benefit in patients with sepsis against a primary composite outcome of mortality, duration of vital support, and length of hospital stay.

## Article Information

### Acknowledgments

Tissue samples were provided by the Imperial College Healthcare National Health Service (NHS) Trust Tissue Bank.

### Sources of Funding

This work was predominantly supported by the British Heart Foundation (grant PG/14/14/30690 to A.J. Hobbs). Tissue samples were provided by the Imperial College Healthcare NHS Trust Tissue Bank. D.B. Antcliffe, A.C. Gordon, and the Imperial Tissue Bank are supported by the National Institute for Health and Care Research (NIHR) Imperial Biomedical Research Center. The views expressed are those of the authors and not necessarily those of the NHS, the NIHR, or the Department of Health and Social Care.

### Disclosures

A.J. Hobbs is a scientific advisory board member/consultant for PharmaIN Corporation, Bionevix Ltd, and Novo Nordisk. A.C. Gordon received consulting fees from AstraZeneca, Beckman Coulter, and VVB Bio, and speaker fees from Fresenius Kabi. The other authors report no conflicts.

## Supplementary Material
