# Supplementary material for: C-Type Natriuretic Peptide Preserves Vascular and Cardiac Function in Sepsis
Source: Hypertension. 2026 Feb 4;83(6):e25938. doi: 10.1161/HYPERTENSIONAHA.125.25938 (PMC13189390; doi:10.1161/HYPERTENSIONAHA.125.25938)
Supplement: Supplementary file 1 [file hyp-83-e25938-s001.pdf]

C-type natriuretic peptide preserves vascular and cardiac function in sepsis

[1]Amie J. Moyes, [1]Claire Sand, [1]Leanne Young, [1]C. Pérez-Ternero, [1]Aemun T. Salam
[1]Reshma S. Baliga, [1]Shireen Mohammad, [2]David B. Antcliffe, [2]Anthony C. Gordon, [1]Aisah A. Aubdool, & [#1]Adrian J. Hobbs

[#]Author for correspondence at the above address:
Email: a.j.hobbs@qmul.ac.uk
Tel: +44 (0)207 882 5778

SUPPLEMENTARY INFORMATION

SUPPLEMENTARY METHODS

Models of endotoxemia and sepsis

Mice (16-20 weeks old; both sexes) were injected intraperitoneally with 12.5mg/kg Lipopolysaccharide (LPS [*Salmonella enterica* serotype *Typhimurium*]; Sigma Aldrich, UK) to induce endotoxemia. Our previous studies indicate that this dose of LPS causes acute hemodynamic and cardiovascular changes associated with the early inflammatory phase of sepsis[1]. The caecal ligation and puncture (CLP) model was used to induce polymicrobial sepsis as this closely resembles the characteristics of intra-abdominal sepsis in humans[2]. CLP was performed as previously described[3]. Briefly, the cecum was ligated below the ileocecal valve (1.5cm from the end) followed by puncturing at either end with a 18G needle. 1mm of feces was squeezed out the puncture holes and then the cecum was returned to the abdomen in its normal anatomical position. Normal saline (20ml/kg) was administered directly after surgery for fluid resuscitation. Antibiotics (Imipenem/Cilastin; 20 mg/kg dissolved in the resuscitation fluid) and an analgesic (buprenorphine; 0.05 mg/kg body weight) were administered at 6 h and 18 h after surgery. Mice were monitored for 24 hours following treatment or surgery and a murine sepsis scoring system designed by Shrum *et al* 2014[4] was used to evaluate the severity of response to LPS or CLP. This score incorporates different variables including appearance, level of consciousness, activity, response to stimulus, eye health, respiration rate and quality. Body temperature was taken using a rectal probe.

Measurement of cardiovascular hemodynamic

Radiotelemetry was used to monitor blood pressure and heart rate following treatment with LPS, as previously described[5]. Briefly, an HDX-11 transmitter (Data Sciences International, USA) was implanted with the blood pressure catheter inserted into the aortic arch via the left carotid artery. Animals were recovered for 10 days before cardiovascular monitoring began. Data was acquired for 2 minutes every 15 minutes and an average reading calculated for each time point (DataArt Acquisition System). Baseline recordings were taken for 2 hours prior to injection with LPS during a period when the mice were inactive so that a stable baseline average could be calculated. Data are expressed as the absolute change from baseline.

Echocardiography was performed to assess heart function before and 24hr after LPS/CLP. M-mode echocardiography was performed using a VisualSonics Vevo 3100 ultrasound system and a 30 MHz transducer (Visualsonics, Amsterdam, The Netherlands). Mice were anesthetized (1.5% isoflurane in O_2) and body temperature maintained at 37°C. Ejection fraction (EF%) was determined from short axis M-mode traces and calculated as follows: EF% = (100 x [LV Vol;d – LV Vol;s/LV Vol;d]). Values were averaged from 3 beats from 3 separate M-mode traces. Pulse wave doppler measurements of blood flow through the mitral valve were taken by scanning the heart in the apical four chamber view. This waveform was used to calculate the left ventricle isovolumetric relaxation time (IVRT) and the myocardial performance index (M.P.I = isovolumetric relaxation time [IVRT] + isovolumetric contraction time [IVCT]/ Aortic ejection time [AET]). Left ventricular posterior wall thickness (LVPW) was measured from short-axis M-mode images.

Measurement of peripheral blood flow

Non-invasive laser doppler imaging (Moor MK2 infrared wavelength high resolution scanner, Moor Instruments Ltd, Devon, UK) was used to assess blood flow in the ear and hindlimb (immobilized with double-sided adhesive tape to avoid movement artifacts) at baseline and 24hr post LPS treatment in anaesthetized mice (2% isofluorane in O_2). The following acquisition modes and settings were used: image scan area 3.7 x 2.6cm; scan distance 34cm; scan speed 4ms/pixel; scan resolution 256 x 153 pixels; flux palette set at 0–700 for ear and 0–5,000 for the hindlimb/paw; background threshold 50 flux units. Blood flow was recorded in flux units and the percentage change from baseline calculated to allow for differences in scanning position, ear/paw size and shape between animals. In some experiments, mice were implanted with osmotic minipumps (1002; Alzet) containing CNP (0.2

mg/kg/day; s.c.) 7 days prior to LPS inoculation to explore if the differences observed in knockout animals could be reversed by pharmacological delivery of the peptide.

Vascular reactivity

The functional reactivity of aorta and mesenteric arteries isolated from naïve and LPS treated animals were assessed by organ bath studies[5]. Concentration-response curves to the vasoconstrictor U46619 (thromboxane mimetic; 1nM-1μM) were constructed in order to assess the function of the contractile apparatus in each vessel. Endothelial function was evaluated by stimulating pre-contracted vessels (using an EC_{50} concentration of U46619) with the endothelium-dependent vasodilator acetylcholine (ACh; 1nM-1μM) and smooth muscle function determined with the NO donor spermine-NONOate (Sper-NO; 1nM-10μM) or atrial natriuretic peptide (ANP; 0.1nM-100nM) . Relaxation is expressed as the percentage change in tension elicited by ACh, with 100% representing maximal relaxation to the baseline resting tension of an unstimulated vessel.

Vascular permeability

A Miles assay was used to assess vascular extravasation as previously described[6]. Evans blue (120 μg/kg; Sigma, UK) was injected intravenously 24 hrs after LPS inoculation. 45 min later, mice were perfused with saline at 70mmHg to remove excess dye from the circulation. Organs were removed, cut into small pieces and incubated in formamide overnight at 55°C to extract the Evans blue. Samples were centrifuged at 10,000g for 20 min and the absorbance of the supernatant was measured at 620 nm using a plate reader. The Evans blue content of each tissue was calculated using a standard curve and expressed as ng Evans blue per mg tissue.

Gene expression

Total RNA was extracted from tissues using the RNeasy Fibrous Tissue Mini Kit (Qiagen, Germany) according to manufacturer instructions. 1000ng RNA from the heart and 500ng from aorta was converted to cDNA using the High Capacity cDNA Reverse Transcription kit (Applied Biosystems) according to manufacturer instructions. The cDNA products were stored at -20°C for qPCR analysis. Quantitative real-time PCR was performed using 10ng of cDNA from heart and aorta tissue with gene-specific primers (0.25μM final concentration; ***Table S3***) and Power Up SYBR Green mastermix (Applied Biosystems). Samples were run on a Biorad CFX96 real-time C1000 touch system. mRNA levels were normalized to RPL-19 (housekeeping gene) and relative expression is represented as a fold change of WT (naïve) or WT (LPS) controls using the $2^{-\Delta\Delta CT}$method, where ΔΔCT is the fold change relative to WT levels. All reactions were performed in duplicate.

Histology

Heart and kidney samples were fixed in formalin for 24hrs and transferred into 70% ethanol before embedding in paraffin blocks. 5μm sections were cut and stained with hematoxylin and eosin (H & E) with an automated Leica auto stainer XL system (Leica Biosystems, UK) by Barts Cancer Institute Pathology Services (Queen Mary University of London, UK). Immunofluorescent staining was performed by dewaxing and rehydrating the samples followed by antigen retrieval (11min in microwave) using a citrate-based solution (pH6.0, Vector Labs). Sections were then washed and permeabilized for 10 min in 0.1% Triton X-100 at room temperature for 15 min followed by incubation with blocking solution (5% goat serum in PBS) for 1h at room temperature. Heart sections were subsequently incubated with primary antibodies against Connexin-43 (1:50, sc-271837, Santa Cruz) or MAC-2 (1:1000, CL8942AP, Cedarlane) in 1% goat serum solution overnight at 4°C. After primary antibody incubation, sections were washed and incubated with appropriate Alexa Fluor-coupled secondary antibodies (1:500, Molecular Probes) for 1h at room temperature. Sections stained with Connexin-43 were co-incubated with a directly conjugated Alexa Fluor 633 Wheat Germ Agglutinin antibody (WGA; 1:500, W32466, Invitrogen) when the secondary antibody was applied. Nuclei were counterstained with DAPI (1:1000) for 10 min. Sections were thoroughly

washed with PBS before mounting them with ProLong mounting media (ThermoFisher Scientific, UK). Sections of kidney were stained with Periodic Acid-Schiff (PAS) by Barts Cancer Institute Pathology Services (Queen Mary University of London, UK). Histological quantification of renal injury was performed at x40 magnification with 20 glomeruli per section analyzed from 6 animals per group by 2 observers. Glomerular damage was defined by decreased Bowman's space, occlusion of capillaries within the Bowman's capsule and inflammatory infiltrates as described in previous studies of LPS induced acute kidney injury[7,8]. The glomeruli were assessed using the following scoring system: 1 = normal or very mild injury with reduced capillaries visible in few glomeruli ; 2= moderate damage with a reduced number of capillaries in a few glomeruli, mild inflammation, <50% reduction in Bowman's space; 3 = large number of glomeruli with reduced capillaries, moderate inflammation, 50% - 75% reduction in Bowman's space; 4 = extensive damage with reduced number of capillaries observed in most glomeruli, 75%-100% reduction in Bowman's space, extensive inflammatory cell infiltrates.

REFERENCES

1. Panayiotou CM, Baliga R, Stidwill R, Taylor V, Singer M, Hobbs AJ. Resistance to endotoxic shock in mice lacking natriuretic peptide receptor-A. *Br J Pharmacol*. 2010;160:2045-2054.
2. Toscano MG, Ganea D, Gamero AM. Cecal ligation puncture procedure. *J Vis Exp*. 2011.
3. Chen J, Kieswich JE, Chiazza F, Moyes AJ, Gobbetti T, Purvis GS, Salvatori DC, Patel NS, Perretti M, Hobbs AJ, et al. IkappaB Kinase Inhibitor Attenuates Sepsis-Induced Cardiac Dysfunction in CKD. *J Am Soc Nephrol*. 2017;28:94-105.
4. Shrum B, Anantha RV, Xu SX, Donnelly M, Haeryfar SM, McCormick JK, Mele T. A robust scoring system to evaluate sepsis severity in an animal model. *BMC Res Notes*. 2014;7:233.
5. Moyes AJ, Khambata RS, Villar I, Bubb KJ, Baliga RS, Lumsden NG, Xiao F, Gane PJ, Rebstock AS, Worthington RJ, et al. Endothelial C-type natriuretic peptide maintains vascular homeostasis. *J Clin Invest*. 2014;124:4039-4051.
6. Perez-Ternero C, Pallier PN, Tremoleda JL, Delogu A, Fernandes C, Michael-Titus AT, Hobbs AJ. C-type natriuretic peptide preserves central neurological function by maintaining blood-brain barrier integrity. *Front Mol Neurosci*. 2022;15:991112.
7. Stasi A, Franzin R, Sallustio F, Scagliotti A, Cappello P, Squiccimarro E, Caggiano G, Losapio R, Campioni M, Castellaneta A, et al. mTOR Inhibition limits LPS induced acute kidney injury and ameliorates hallmarks of cellular senescence. *Sci Rep*. 2025;15:9635.
8. Li G, Wei W, Suo L, Zhang C, Yu H, Liu H, Guo Q, Zhen X, Yu Y. Low-Dose Aspirin Prevents Kidney Damage in LPS-Induced Preeclampsia by Inhibiting the WNT5A and NF-kappaB Signaling Pathways. *Front Endocrinol (Lausanne)*. 2021;12:639592.

SUPPLEMENTARY TABLES

	Controls	Septic
Age (years)	62.5 (48.75-71.25)	62.5 (43-78)
Sex (Male:Female)	5:7	7:7
MABP (mmHg)	75 (66.25-80.0)	70.5 (59.75-74.75)
Total SOFA Score<br>**SOFA Respiratory**<br>**SOFA Cardiovascular**<br>**SOFA Liver**<br>**SOFA Coagulation**<br>**SOFA CNS**<br>**SOFA Renal**	14 (12-14.2)<br>2 (1.8-3)<br>3 (2.2-4)<br>0 (0-0.2)<br>4 (4-4)<br>4 (4-4)<br>0 (0-0)	8.5 (8-10.25)<br>4 (4-4)<br>4 (3-4)<br>0 (0-1)<br>0 (0-0)<br>0 (0-2)<br>0 (0-1)
Ischemic Heart Disease	0/12	0/14
NYHA IV (Heart Failure)	0/12	0/14
Severe COPD	0/12	1/14
Chronic renal failure	0/12	0/14
Cirrhosis	0/12	0/14
Cancer	2/12	3/14
Diabetes	1/12	0/14

Table S1. Control and septic patient demographics. Abbreviations: Mean Arterial Blood Pressure (MABP), Sequential Organ Failure Assessment score (SOFA), New York Heart Association (NYHA) Classification, Chronic Obstructive Pulmonary Disease (COPD). Median + IQR.

	WT	ecCNP$^{-/-}$	P value (v WT)	cmCNP$^{-/-}$	P value (v WT)	NPR-C$^{-/-}$	P value (v WT)
**Ear flux**	141.5±3.2	147.5±5.4	P=0.610	143.3±7.0	P=0.989	136.2±2.7	P=0.699
**Hindlimb flux**	1550±78	1744±99	P=0.339	1387±99	P=0.632	1395±111	P=0.535

Table S2. Baseline ear and hindlimb flux in wild type (WT), endothelium-restricted C-type natriuretic peptide knockout (ecCNP$^{-/-}$) or global natriuretic peptide receptor-C knockout (NPR-C$^{-/-}$) mice. Data is represented as mean±SEM. n=6. Statistical analysis by one-way ANOVA with Dunnett's post-hoc test. Each statistical comparison undertaken has an assigned *P* value (adjusted for multiplicity).

**Target Gene**	**Forward Primer Sequence (5'-3')**	**Reverse Primer Sequence (5'-3')**
CNP (*Nppc*)	CCAACGCGCGCAAATACAAA	GCACAGAGCAGTTCCCAATC
NPR-B (*Npr2*)	AACGGGCGCATTGTGTATATCT	TCAGGATTTGGGGGTTCTCG
NPR-C (*Npr3*)	CTTGGATGTAGCGCACTATGTC	CACAAGGACACGGAATACTC
Rpl19 (*Rpl19*)	TTGGCGATTTCATTGGTCTCA	GCTTGCCTCTAGTGTCCTCC
Connexin 43 (*Gja1*)	ACAGGTCTGAGAGCCCGAAC	TTACAGCGAAAGGCAGACTGT
ZO-1 (*Tjp1*)	GCGCGGAGAGAGACAAGATGT	CAACTCGGTCATTTTCCTGTAGC
VE-Cadherin (*Cdh5*)	GCTCACGGACAAGATCAGCTC	ACTTAGCATTCTGGCGGTTCA
IL-6 (*Il6*)	TCGTGGAAATGAGAAAAGAGTTGTG	ACTCCAGAAGACCAGAGGAAA
iNOS (*Nos2*)	GGTGAAGGGACTGAGCTGTT	GTCATCTTGTATTGTTGGGCTGA
CCL-2 (*Ccl2*)	GAAGCTGTAGTTTTTGTCACCA	TTCCTTCTTGGGGTCAGCAC
IL-1β (*Il1b*)	TGCCACCTTTTGACAGTGATG	TGATGTGCTGCTGCGAGATT
TNFα (Tnf)	ACCCTCACACTCACAAACCA	TTGAGATCCATGCCGTTGGC

Table S3. Primer sequences used for qPCR. Gene symbols are included in italics.

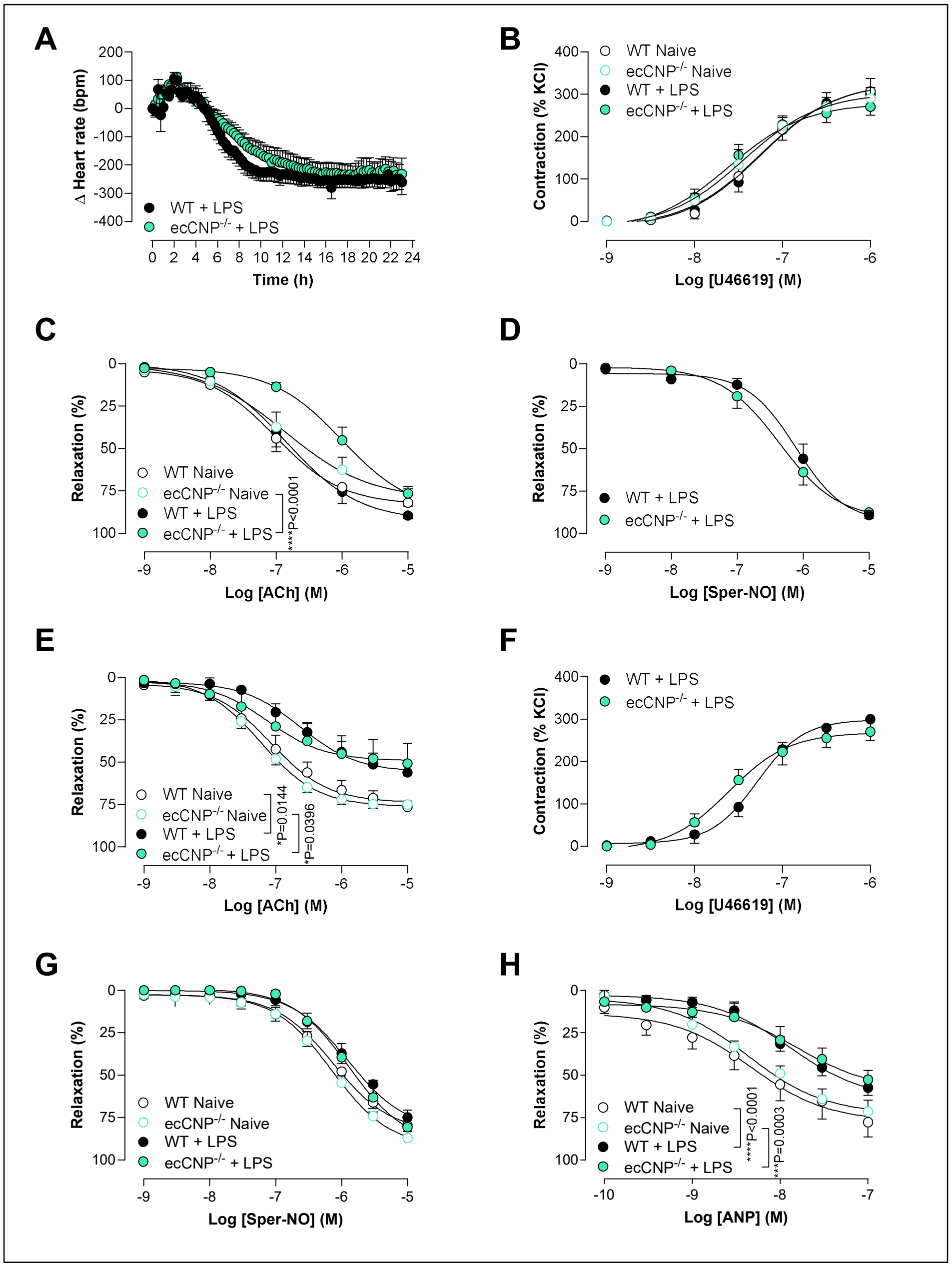

Figure S1. Change (Δ) in heart rate measured by radiotelemetry for 24hr after LPS administration (12.5mg/kg, i.p.; ***A***), and comparison of vasoconstrictor responses to U46619 and vasodilator responses to acetylcholine (ACh), spermine-NONOate (Sper-NO) and atrial natriuretic peptide (ANP) in mesenteric arteries (***B,C,D***) or aorta (***E,F,G,H***) from wild type (WT) or endothelium-restricted C-type natriuretic peptide knockout ($ecCNP^{-/-}$) mice in the absence and presence of LPS administration (12.5mg/kg; i.p.; 24hr). Data are represented as mean±SEM. *n*=6-12. Statistical analysis by two-way ANOVA (***A-H***). Only statistically-significant P values are indicated for clarity.

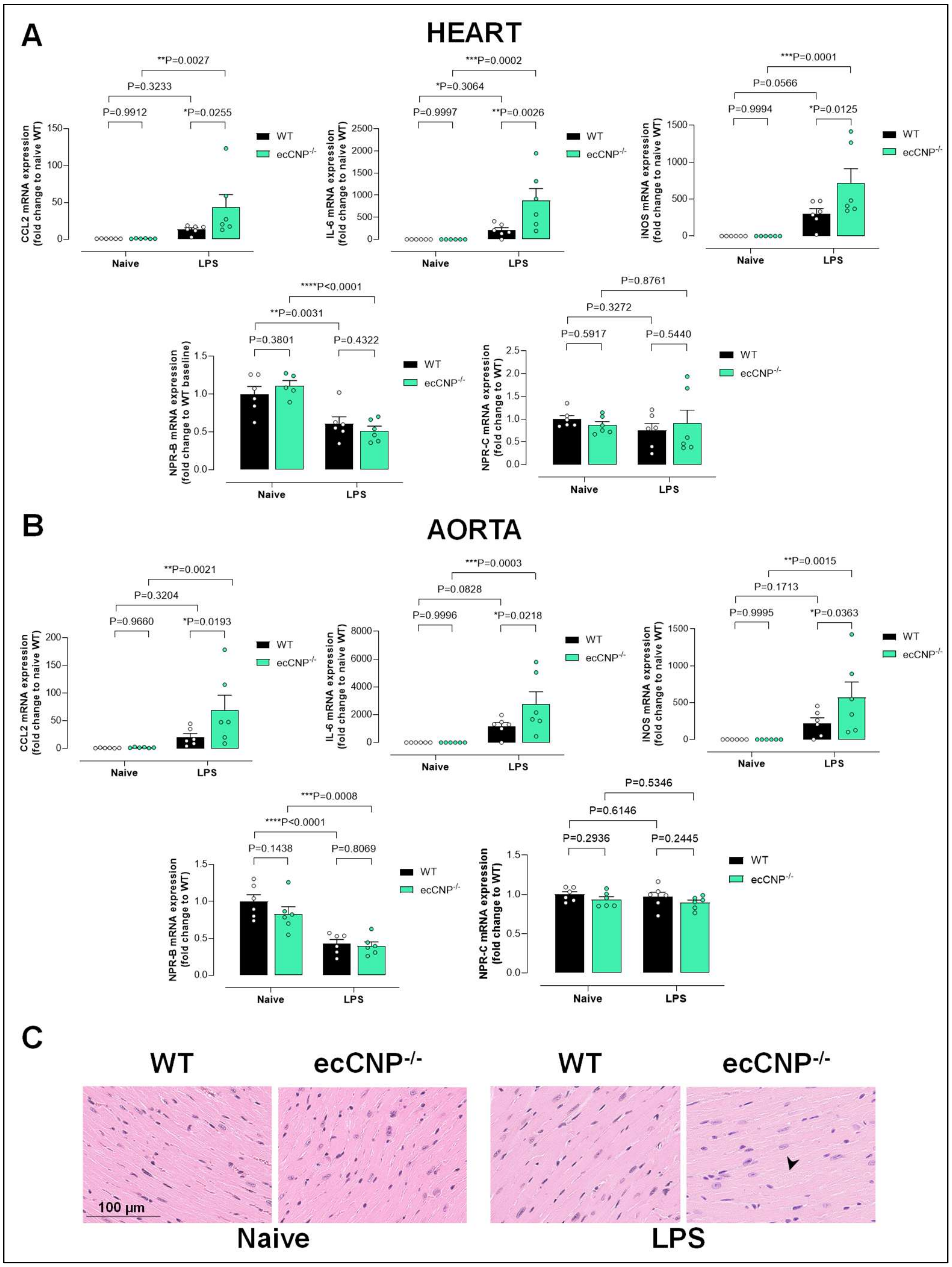

Figure S2. Raw data of the mRNA expression of the inflammatory markers CCL-2, IL-6, iNOS, and natriuretic peptide receptors NPR-B and NPR-C in the heart (***A***) and aorta (***B***) of wild type (WT) and endothelium-restricted C-type natriuretic peptide knockout (ecCNP$^{-/-}$) mice in the absence and presence of LPS administration (12.5mg/kg; i.p.; 24hr). H&E staining of LPS-treated hearts highlighting areas of myocytolysis (***C***; black arrow). Data are represented as mean±SEM. *n*=6. Statistical analysis by two-way ANOVA with Fisher's post hoc test. Each statistical comparison undertaken has an assigned *P* value (adjusted for multiplicity).

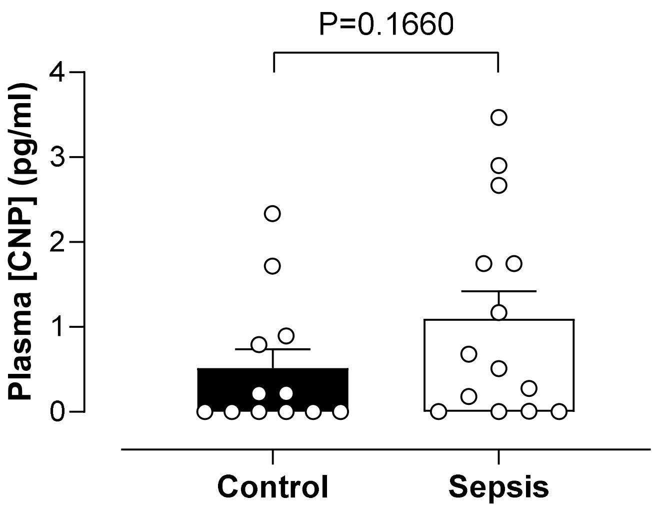

Figure S3. Plasma concentrations of C-type natriuretic peptide (CNP) in healthy control controls and septic patients. Data are represented as mean±SEM *n*=12-*14*. Statistical analysis by unpaired, two-tailed t-test.

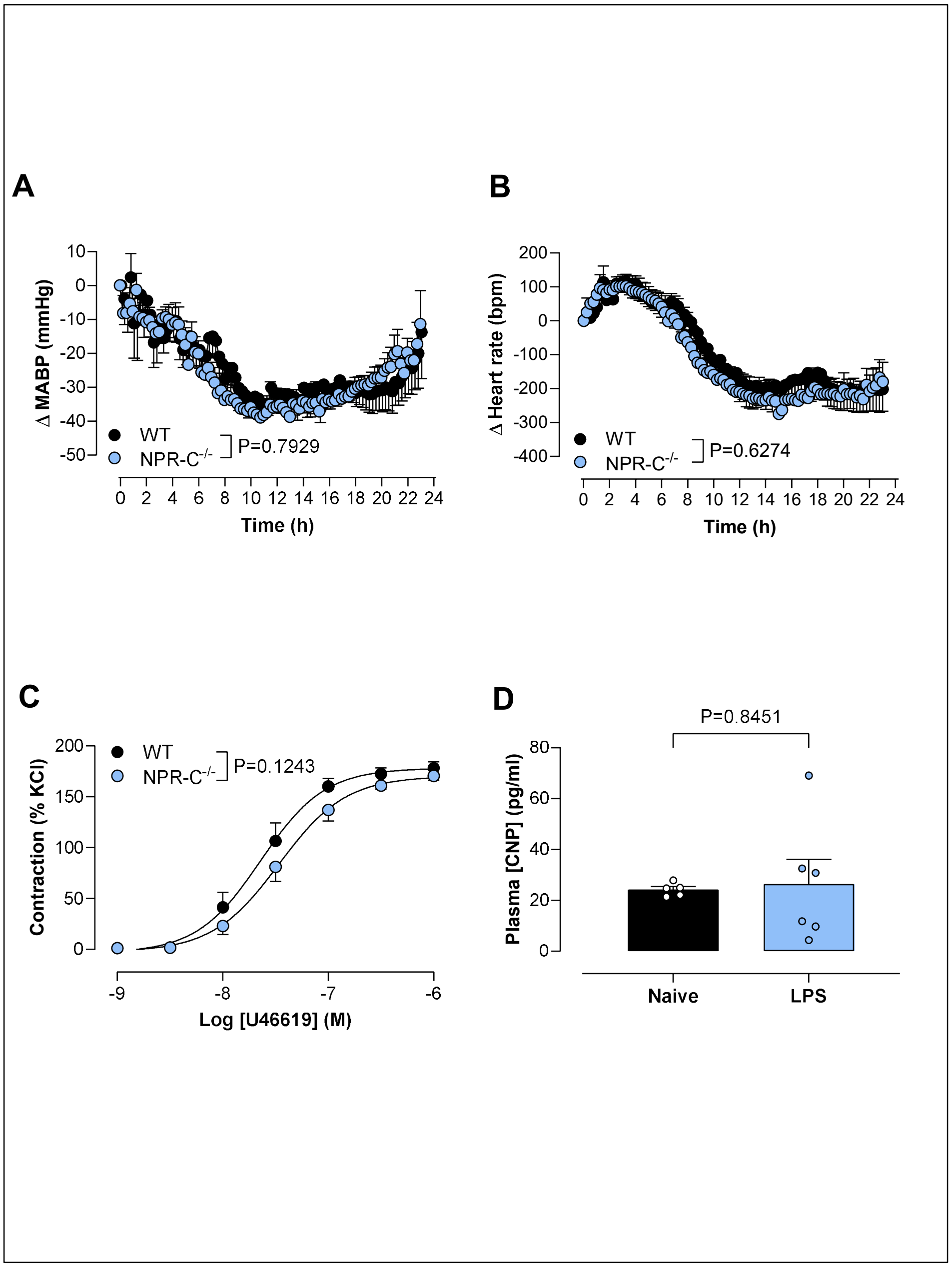

Figure S4. Change (Δ) in mean blood pressure (MABP; ***A***) and heart rate (***B***) measured by radiotelemetry for 24hr, and comparison of vasoconstrictor responses to U46619 in mesenteric arteries from LPS-treated animals in natriuretic peptide receptor (NPR)-C knockout (NPR-$C^{-/-}$) mice and wild type (WT) littermates (***C***) after LPS administration (12.5mg/kg; i.p.). Plasma C-type natriuretic peptide (CNP) concentrations (***D***) in global natriuretic peptide receptor (NPR)-C knockout (NPR-$C^{-/-}$) mice in the absence (naïve) and presence of LPS administration (12.5mg/kg; i.p.; 24hr). Data are represented as mean±SEM. n=6-8. Statistical analysis by two-way ANOVA (***A,B,C***) or unpaired Student's t-test (***D***). Each statistical comparison undertaken has an assigned *P* value (adjusted for multiplicity).

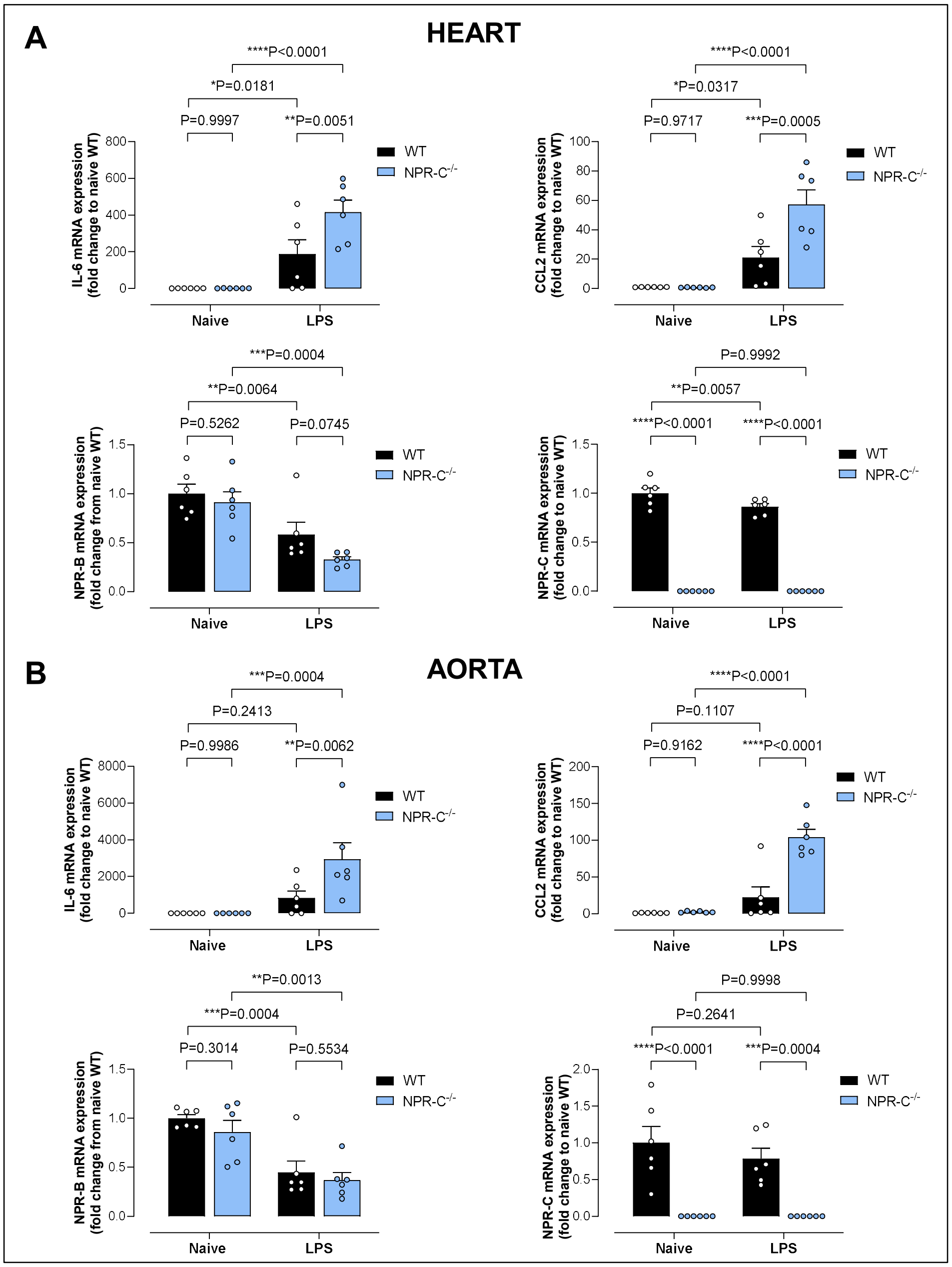

Figure S5. mRNA expression of the inflammatory markers IL-6, CCL-2, and natriuretic peptide receptors NPR-B and NPR-C in the heart (***A***) and aorta (***B***) of naïve and LPS (12.5mg/kg, i.p., 24hr) treated global natriuretic peptide receptor (NPR)-C$^{-/-}$ animals and wildtype (WT) littermates. Data are represented as mean±SEM. n=6. Statistical analysis by two-way ANOVA with Fisher's post hoc test. Each statistical comparison undertaken has an assigned P value (adjusted for multiplicity).

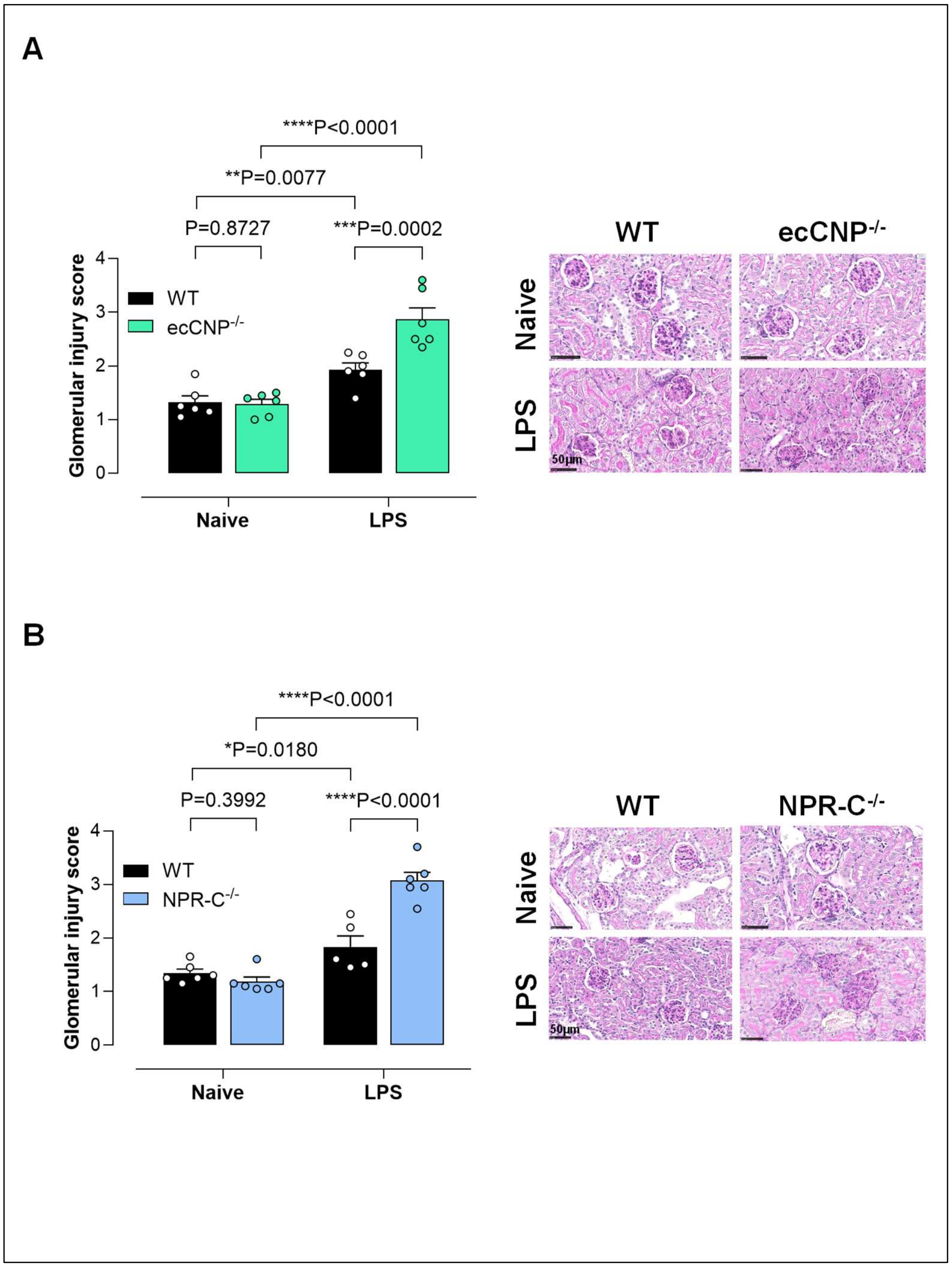

Figure S6. Quantification and representative images of glomerular injury score in wild type (WT) and endothelium-restricted C-type natriuretic peptide knockout (ecCNP$^{-/-}$; ***A***) or global natriuretic peptide receptor (NPR)-C$^{-/-}$ (***B***) mice in the absence and presence of LPS administration (12.5mg/kg; i.p.; 24hr). Data are represented as mean±SEM. *n*=6. Statistical analysis by two-way ANOVA with Fisher's post hoc test. Each statistical comparison undertaken has an assigned *P* value (adjusted for multiplicity).

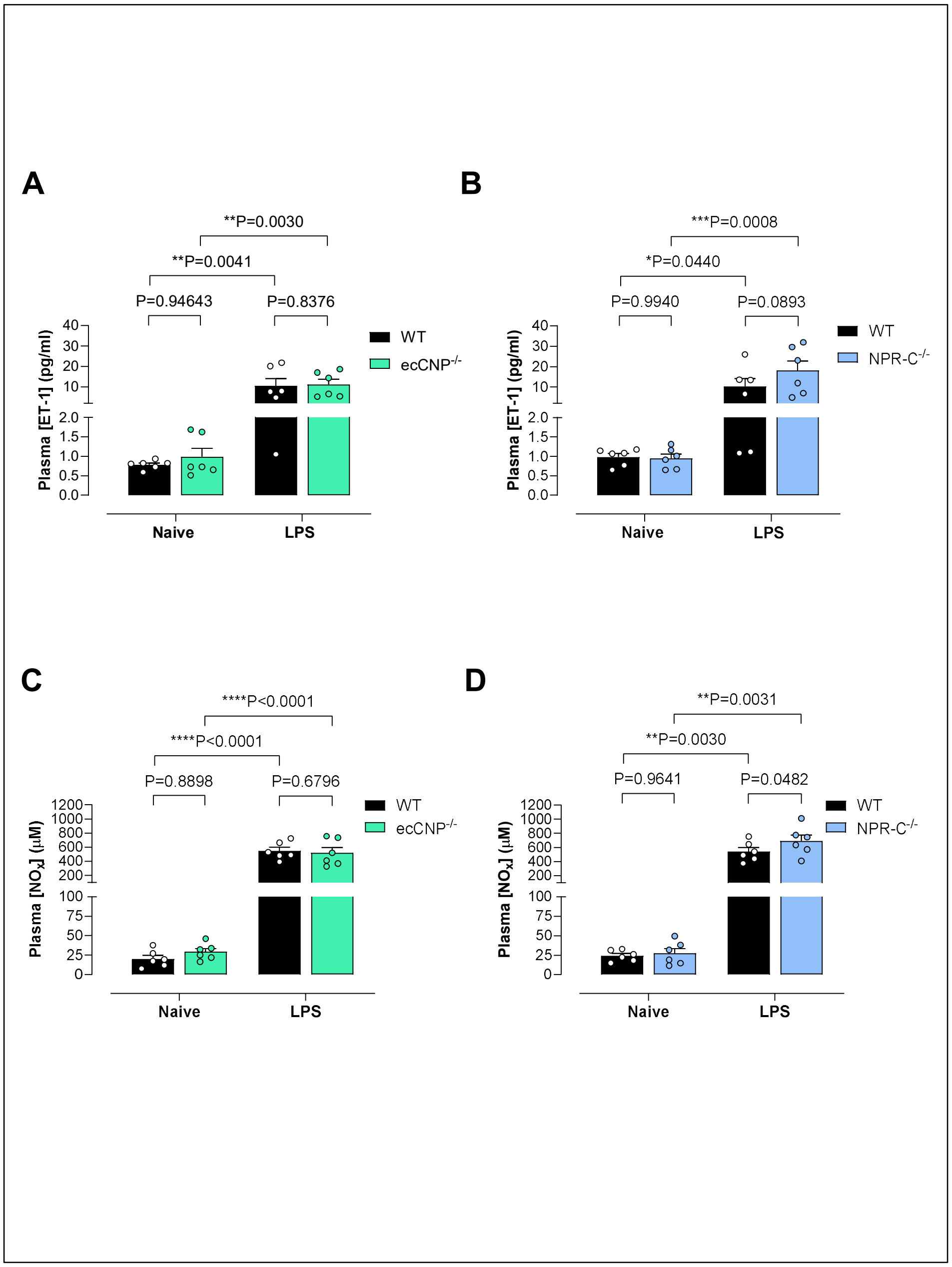

Figure S7. Plasma endothelin (ET)-1 (***A,B***) and inorganic nitrite plus nitrate (NO_x; ***C,D***) in endothelium-restricted C-type natriuretic peptide ($ecCNP^{-/-}$) or global natriuretic peptide receptor (NPR)-C knockout ($NPR\text{-}C^{-/-}$) mice and wild type (WT) littermates in the absence (naïve) and presence of LPS administration (12.5mg/kg; i.p.; 24hr). Data are represented as mean±SEM. n=6. Statistical analysis by two-way ANOVA. Each statistical comparison undertaken has an assigned *P* value (adjusted for multiplicity).

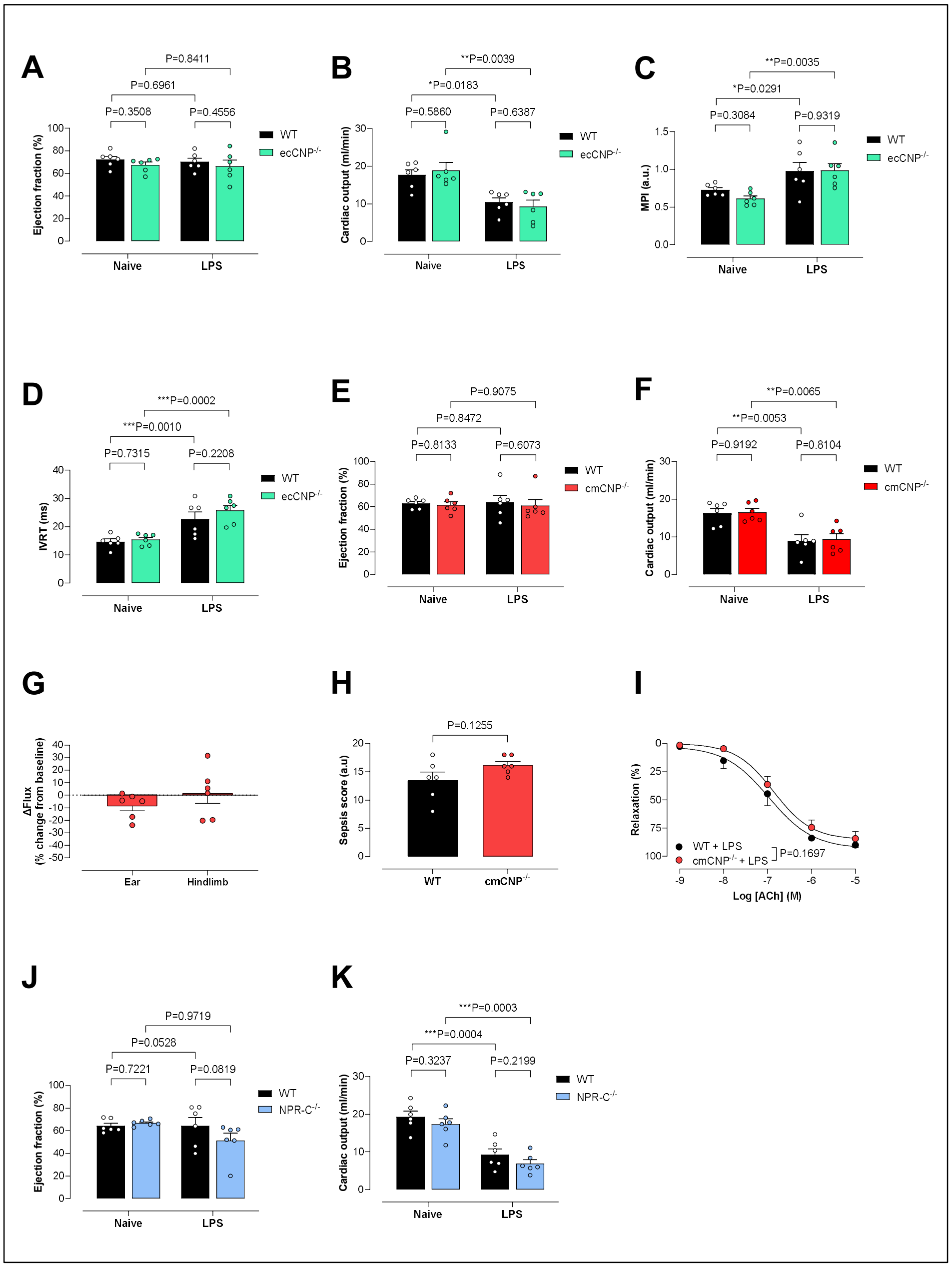

Figure S8. Ejection fraction (***A,E,J***), cardiac output (***B,F,K***), myocardial performance index (MPI, ***C***), isovolumic relaxation time (IVRT; ***D***), change (Δ) in ear and hindlimb blood flow (***G***), sepsis score (***H***), vasorelaxant responses to the endothelium-dependent dilator acetylcholine (ACh; ***I***) in wild type (WT), endothelium-restricted C-type natriuretic peptide knockout (ecCNP$^{-/-}$) (***A-D***) cardiomyocyte-restricted C-type natriuretic peptide knockout (cmCNP$^{-/-}$) (***E-I***) or global natriuretic peptide receptor (NPR)-C knockout (NPR-C$^{-/-}$) mice (***J &K***) in the absence and presence of LPS (12.5mg/kg, i.p., 24hr). Data are represented as mean±SEM. *n*=8. Statistical analysis by two-way ANOVA with Fisher's post hoc test (***A,B,C,D,E,F,I,J,K***) or unpaired Student's t-test (***G,H***). Each statistical comparison undertaken has an assigned *P* value (adjusted for multiplicity).

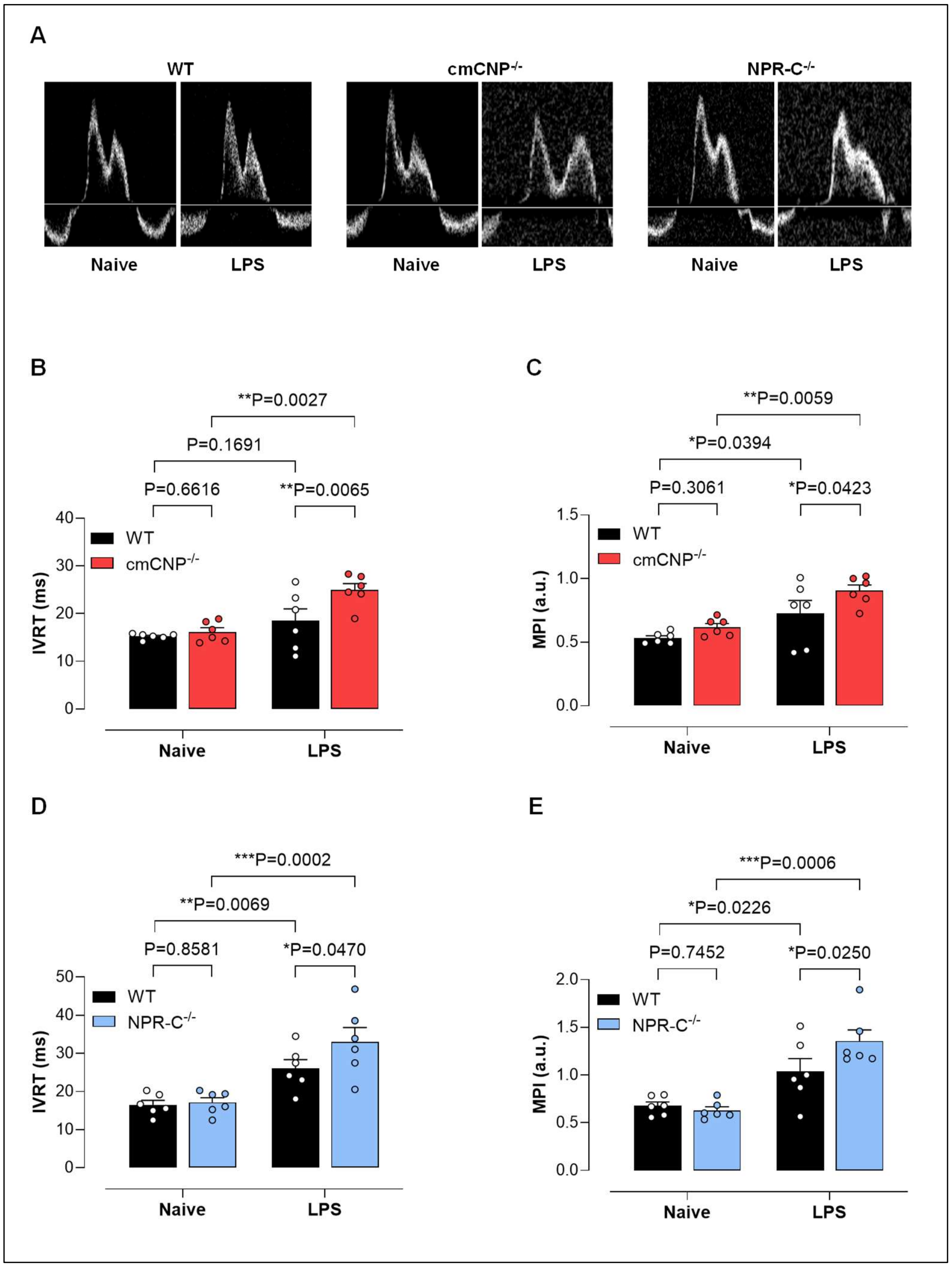

Figure S9. Representative echocardiographic images of mitral valve pulse wave doppler (***A***), isovolumic relaxation time (IVRT; ***B,D***) and myocardial performance index (MPI; ***C,E***) in wild type (WT), cardiomyocyte-restricted C-type natriuretic peptide knockout (cmCNP$^{-/-}$) knockout or global natriuretic peptide receptor (NPR)-C knockout (NPR-C$^{-/-}$) mice in the absence and presence of LPS administration (12.5mg/kg; i.p.; 24hr). Data are represented as mean±SEM. n=6. Statistical analysis by two-way ANOVA with Fisher's post hoc test. Each statistical comparison undertaken has an assigned *P* value (adjusted for multiplicity).

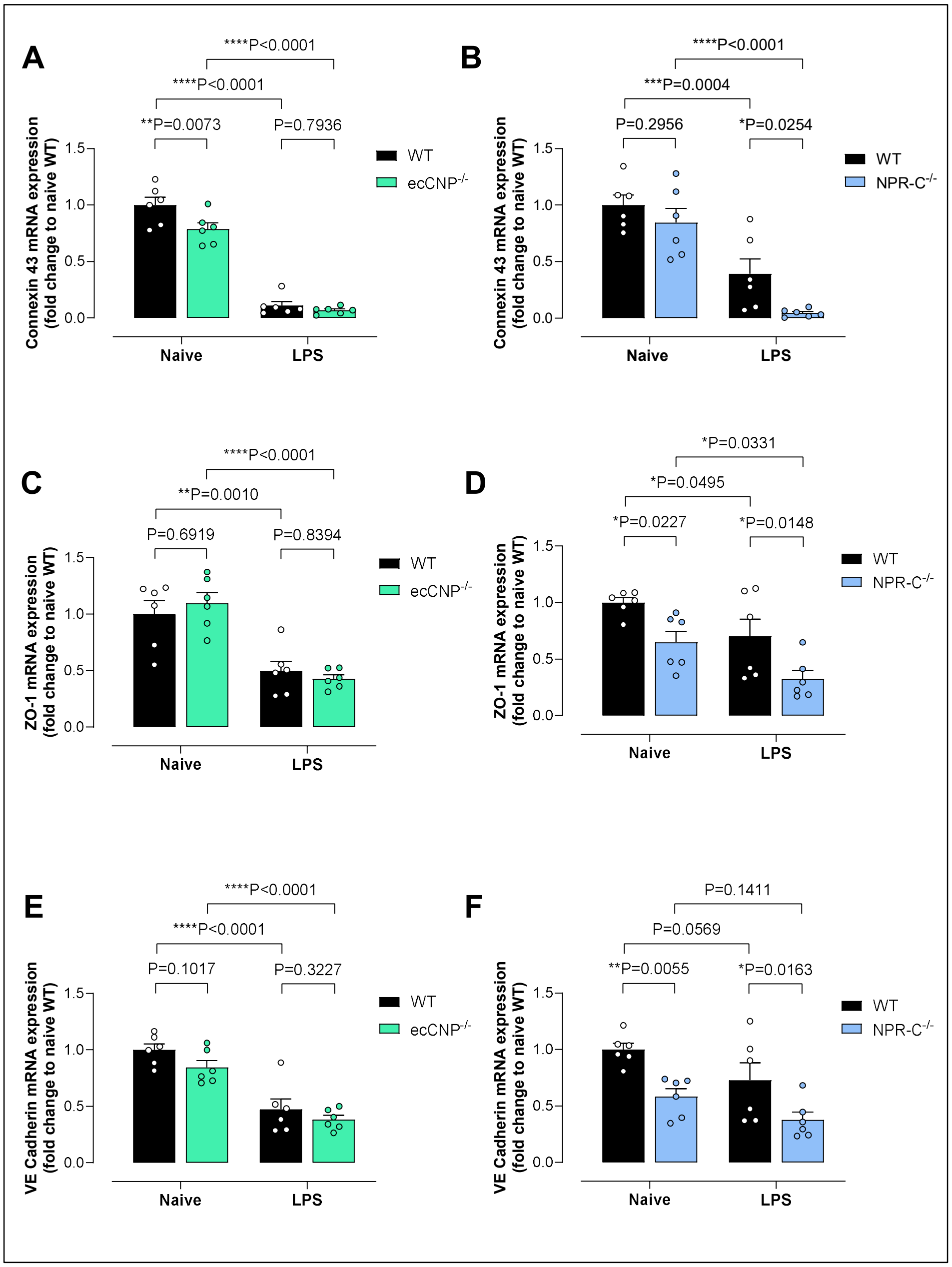

Figure S10. Cardiac connexin 43 (***A, B***), zona occludin 1 (ZO-1; ***C, D***) and vascular endothelial (VE) Cadherin (***E, F***) mRNA expression in the absence (naïve) and presence of LPS (12.5mg/kg/day; i.p) in wild type (WT), endothelium-restricted C-type natriuretic peptide (ecCNP$^{-/-}$; ***A,C,E***) or global natriuretic peptide receptor (NPR)-C knockout (NPR-C$^{-/-}$; ***B,D,F***) mice treated with LPS (12.5mg/kg, i.p., 24hr). Data are represented as mean±SEM. *n*=6. Statistical analysis by two-way ANOVA with Fisher's post hoc test. Each statistical comparison undertaken has an assigned *P* value (adjusted for multiplicity).

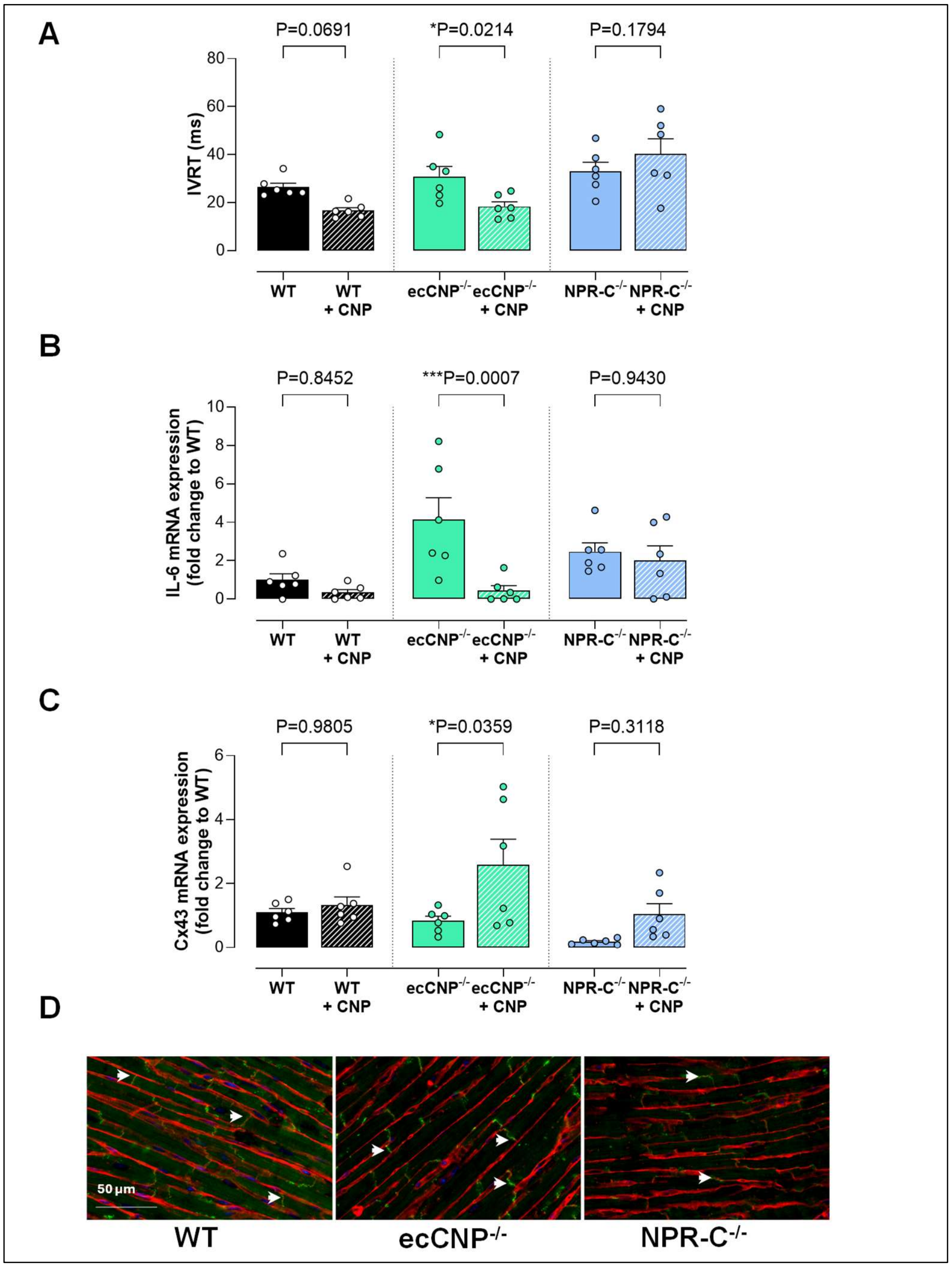

Figure S11. Isovolumetric relaxation time (IVRT; ***A***), and mRNA expression of interleukin (IL)-6 (***B***) and Connexin 43 (***C***), with representative images of Connexin-43 immunostaining (***D***) (Cx43, green; cell membrane marker wheat germ agglutinin, WGA, red) in LPS (12.5mg/kg, i.p., 24hr)-treated endothelium-restricted C-type natriuretic peptide knockout ($ecCNP^{-/-}$) and global natriuretic receptor (NPR)-C knockout ($NPR\text{-}C^{-/-}$) mice and wild type (WT) littermates in the absence and presence of CNP (0.2mg/kg/day; s.c). Data are represented as mean±SEM. *n*=6. Statistical analysis by two-way ANOVA with Tukey's post hoc test. Each statistical comparison undertaken has an assigned *P* value (adjusted for multiplicity).
